# GC content of vertebrate exome landscapes reveal areas of accelerated protein evolution

**DOI:** 10.1186/s12862-019-1469-1

**Published:** 2019-07-16

**Authors:** R. Huttener, L. Thorrez, T. in’t Veld, M. Granvik, L. Snoeck, L. Van Lommel, F. Schuit

**Affiliations:** 1 Gene Expression Unit, Dept of Cellular and Molecular Medicine, KU Leuven, Leuven, Belgium; 2 Tissue Engineering Laboratory, Dept of Development and Regeneration, KU Leuven, Kortrijk, Belgium

**Keywords:** Exome landscapes, GC biased gene conversion, Comparative analysis, Vertebrates, GC content, Amino acid composition, Protein evolution

## Abstract

**Background:**

Rapid accumulation of vertebrate genome sequences render comparative genomics a powerful approach to study macro-evolutionary events. The assessment of phylogenic relationships between species routinely depends on the analysis of sequence homology at the nucleotide or protein level.

**Results:**

We analyzed mRNA GC content, codon usage and divergence of orthologous proteins in 55 vertebrate genomes. Data were visualized in genome-wide landscapes using a sliding window approach. Landscapes of GC content reveal both evolutionary conservation of clustered genes, and lineage-specific changes, so that it was possible to construct a phylogenetic tree that closely matched the classic “tree of life”. Landscapes of GC content also strongly correlated to landscapes of amino acid usage: positive correlation with glycine, alanine, arginine and proline and negative correlation with phenylalanine, tyrosine, methionine, isoleucine, asparagine and lysine. Peaks of GC content correlated strongly with increased protein divergence.

**Conclusions:**

Landscapes of base- and amino acid composition of the coding genome opens a new approach in comparative genomics, allowing identification of discrete regions in which protein evolution accelerated over deep evolutionary time. Insight in the evolution of genome structure may spur novel studies assessing the evolutionary benefit of genes in particular genomic regions.

**Electronic supplementary material:**

The online version of this article (10.1186/s12862-019-1469-1) contains supplementary material, which is available to authorized users.

## Background

Base composition of vertebrate genomes fluctuates in function of the regional position in the different chromosomes [1]. At the megabase scale, the change in GC content in continuous DNA sequence, without discrimination between introns, exons, intergenic sequence, is called isochores [2, 3]. The function of this structural variability has been debated since decades. The idea of an adaptation in warm-blooded vertebrates was abandoned as isochores are also prominent in cold blooded reptiles [3]. More recently, GC-biased gene conversion (gBGC) was proposed to be a mechanism that could accomplish the massive accumulation of G and C bases in certain chromosomal regions. The key event occurs during DNA repair after meiotic crossing over: when the repair enzymes encounter ambiguous base-pairs, a tiny advantage favoring G or C over A and T is present [4, 5]. Although this bias causes miniscule effects on base composition per generation, gBGC is thought to lead to significant fluctuations in GC content over deep evolutionary time [5–7]. While gBGC has been most studied in primate genomes [8–11], it was demonstrated to affect regional base composition of other eukaryotes comprising mammals [12, 13], reptiles [14], birds [15, 16] and plants [17]. As gBGC also affects base composition of the coding sequence, it is considered a relevant driving force for protein evolution [9, 18, 19]. A review of human diseases as a consequence of fixation of a deleterious allele by gBGC is given in [20]. Another interesting aspect of gBGC is that the bias to alter base composition is also directed to particular chromosomal regions. At least one recombination event per chromosome is required to allow proper segregation of chromosomes. Therefore, small chromosomes over time are more affected by gBGC than large chromosomes. Local areas with increased likelihood for recombination (hot spots) are correlated with elevated GC content [4, 8, 11]. In primates, recombination hot spots accumulating GC bases are mostly seen at the subtelomeric regions of chromosomes [8].

The fluctuation of genomic GC content over deep time and regional changes within genomes were correlated to codon usage, both in prokaryotes [21] and protists [22]. This correlation is positive for amino acids encoded by GC-rich codons (glycine, alanine, arginine and proline - GARP) and negative for residues encoded by AU-rich codons (phenylalanine, tyrosine, methionine, isoleucine, asparagine and lysine - FYMINK).

Together, research of gBGC in eukaryotic organisms indicates that the genomic position of a gene, being localized either in GC-depleted or GC-enriched DNA, has important evolutionary consequences [5]. Given this importance, we have assessed possibilities to compare regional fluctuations of base composition of predicted mRNAs, amino acid composition of predicted proteins and protein divergence over a broad range of vertebrate species in a comprehensive genome-wide display. We propose a sliding window approach to visualize exome landscapes, either at the level of base composition, amino acid composition or protein divergence and use this tool for comparisons between different vertebrate species. We anticipate that this will be useful to assess the macro-evolutionary significance of lineage specific and common vertebrate events.

## Results

### Construction of genome-wide exome landscapes of GC content

We performed a comparative analysis among 55 different vertebrates (a list of the used species is given in Additional file 1: Table S1). The species in our analysis were distributed over the main vertebrate clades in the tree of life. From the fish lineages, however, we included only non-teleost species to avoid effects of the teleost genome duplication event [23]. For birds and non-avian reptiles we selected genomes with the highest number of annotated genes. For all analyzed species, we assessed the GC content of predicted mRNAs from a set of 15,824 vertebrate protein-encoding genes.

**Fig. 1:**
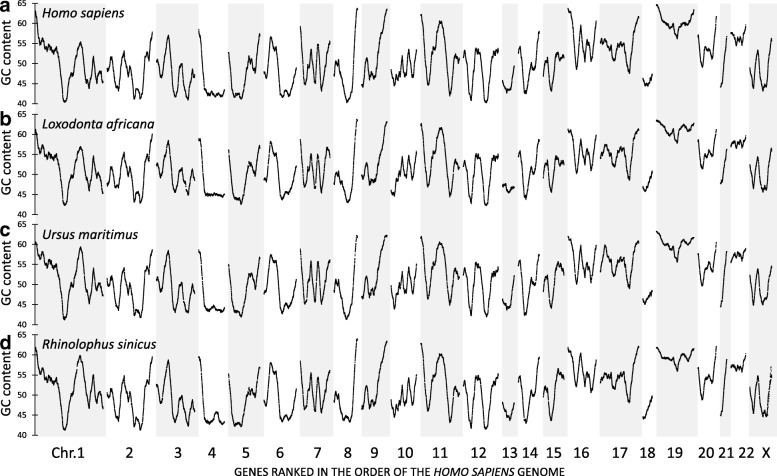
Genome-wide landscapes of base composition of predicted mRNAs from four placental mammals. GC content from a vertebrate gene set of 15,824 predicted mRNAs encoded in the genomes of African elephant (*Loxodonta africana, panel* **b**), polar bear (*Ursus maritimus, panel* **c**) and Chinese rufous horseshoe bat (*Rhinolophus sinicus, panel* ***d***) were compared to human data (panel **a**). Orthologous genes were ranked on the order of the human genome and GC content was calculated using a sliding window approach of a gene and its 100 surrounding genes. All four mammalian species show highly similar patterns of GC content fluctuations

**Fig. 2:**
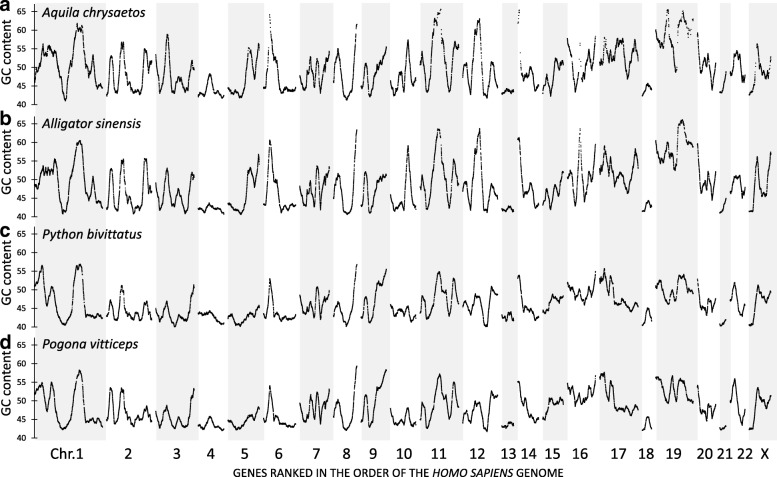
Genome-wide landscapes of base composition of predicted mRNAs from four reptiles. **a*.*** *Aquila chrysaetos canadensis* (golden eagle), **b.** *Alligator sinensis* (Chinese alligator), **c.** *Python bivittatus* (Burmese python), and **d**. *Pogona vitticeps* (central bearded dragon). Genes were ranked on the order of the human genome and GC content was calculated using a sliding window approach of a gene and its 100 surrounding genes. Only minor differences between the landscapes of these four reptiles can be observed, while major differences can be seen compared to the mammalian landscapes of Fig. 1

We calculated the mean GC content of a gene and its 100 neighboring genes (50 upstream and 50 downstream of the gene) and this window was slided in steps of one gene over all positions of the reference genome. This sliding window size provided a good tradeoff between smaller window sizes displaying the erratic nature of the values of individual genes and larger window sizes resulting in excessive smoothing (Additional file 2: Figure S1). Visualization of human genes, when ordered linearly over the human chromosomes, revealed a characteristic landscape with prominent regional peaks and valleys of GC content (Fig. 1a). The landscapes of the GC content of the mRNA transcripts were comparable to those of the whole gene sequences or intron sequence (Additional file 3: Figure S2). However, transcript sequence had a consistently higher GC content than intronic sequence. Also, we verified the minor impact of reducing the total number of 19,971 human protein-encoding genes to a core common vertebrate set of 15,824 protein encoding genes (Additional file 4: Figure S3). The addition of 4147 extra genes did not fundamentally change the fluctuations of the GC content landscape. Finally, we compared the human GC landscape based on the numerical gene order and physical distance on the chromosomes (Additional file 5: Figure S4). We observed narrowing of GC peaks and broadening of valleys in the physical density landscape, which is in agreement with the observation of a higher gene density in GC rich genome areas [24, 25]. Given the robustness over the overall landscape features, we next compared the GC landscapes of human genes with those obtained from the orthologous genes of other placental mammals such as *Loxodonta africana* (African elephant), *Ursus maritimus* (polar bear) or *Rhinolophus sinicus* (Chinese rufous horseshoe bat). To allow direct comparison, the orthologous genes were ordered according to their position on a reference genome (indicated on the graphs). Despite a phylogenic distance of about 100 million years (range 94–102) and numerous synteny breaks between the different mammalian genomes, we found remarkable similarity between the human landscape and the landscapes of the other placental mammals. This counterintuitive result was further extended by the analysis of four genomes that belong to the class of “Reptilia”: *Aquila chrysaetos canadensis (*golden eagle*)*, *Alligator sinensis* (Chinese alligator), *Python bivittatus* (Burmese python) and *Pogona vitticeps* (central bearded dragon). We used the same sliding window approach of calculated transcript GC content, with genes ordered according to the position on the human reference genome. Despite phylogenic distances of nearly 300 million years, the reptilian landscapes shared important characteristic points with each other (Fig. 2) while they all displayed many different peaks and valleys compared to the mammalian counterparts (Fig. 2 versus Fig. 1). Details of these differences can be seen in Additional file 6: Figure S5 where we plot three representative examples of the large amount of pairwise comparisons between a mammalian (*N* = 21) and reptilian (*N* = 29 including birds) landscapes. We found no clear differences in landscapes based on the environmental niche (e.g. terrestrial versus aquatic).

**Fig. 3:**
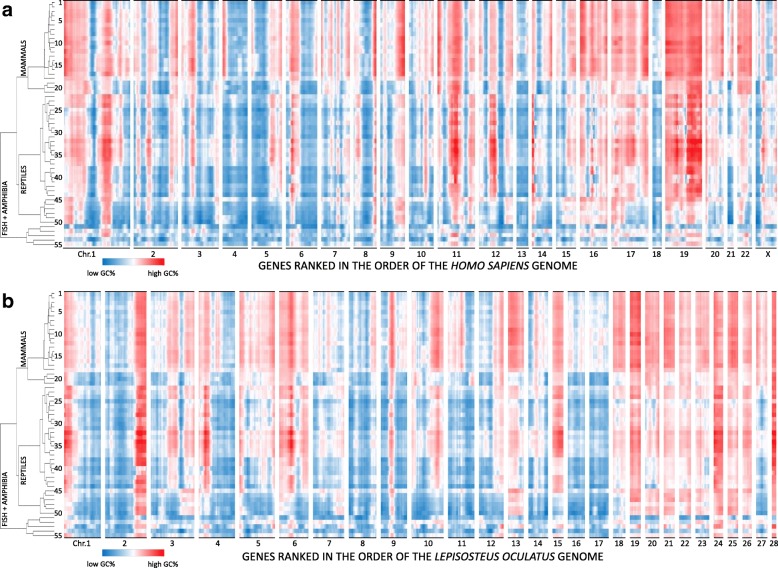
Heat map of genome wide GC landscapes of 55 vertebrate species. Sliding window means of GC content of predicted mRNAs were calculated as in Figs. 1 and 2. Data of 55 vertebrate genomes are presented in a heat map in which the genes were ordered on basis of the *Homo sapiens* (**a**) or *Lepistosteus oculatus* (**b**) reference genome. Vertebrate genomes were ordered on basis of GC similarities and numbered as follows: Mammals (1–21): 1 *Homo sapiens;* 2 *Gorilla gorilla;* 3 *Ursus maritimus;* 4 *Ailuropoda melanoleuca;* 5 *Panthera tigris altaica;* 6 *Loxodonta Africana;* 7 *Rhinolophus sinicus;* 8 *Hipposideros armiger;* 9 *Sus scrofa;* 1*0 Panthera pardus;* 11 *Odocoileus virginianus texanus;* 12 *Physeter catodon;* 13 *Miniopterus natalensis;* 14 *Myotis davidii;* 15 *Myotis brandtii;* 16 *Myotis lucifugus;* 17 *Pteropus vampyrus;* 18 *Mus musculus;* 19 *Phascolarctos cinereus;* 2*0 Sarcophilus harrisii;* 21 *Monodelphis domestica;* Reptiles (22–50): 22 *Tinamus guttatus;* 23 *Calypte anna;* 24 *Columba livia;* 25 *Struthio camelus australis;* 26 *Apteryx australis mantelli;* 27 *Aptenodytes forsteri;* 28 *Aquila chrysaetos canadensis;* 29 *Anas platyrhynchos;* 3*0 Anser cygnoides domesticus;* 31 *Corvus brachyrhynchos;* 32 *Lonchura striata domestica;* 33 *Parus major;* 34 *Pseudopodoces humilis;* 35 *Sturnus vulgaris;* 36 *Gallus gallus;* 37 *Coturnix japonica;* 38 *Alligator sinensis;* 39 *Alligator mississippiensis;* 4*0 Gavialis gangeticus;* 41 *Crocodylus porosus;* 42 *Chelonia mydas;* 43 *Chrysemys picta;* 44 *Pelodiscus sinensis;* 45 *Gekko japonicas;* 46 *Pogona vitticeps;* 47 *Anolis carolinensis;* 48 *Python bivittatus;* 49 *Protobothrops mucrosquamatus;* 5*0 Thamnophis sirtalis;* Fish and amphibia 51–55): 51 *Xenopus tropicalis;* 52 *Nanorana parkeri;* 53 *Lepisosteus oculatus;* 54 *Latimeria chalumnae;* 55 *Callorhinchus milii.* This visual display captures the overall idea of conservation of GC poor and GC rich regions in vertebrate genomes and the appearance of lineage speficic GC accumulation, especially in mammals and in reptiles, including birds

To further integrate the information of the sliding window GC landscapes of 55 vertebrate genomes, we constructed a heat map in which the genes were ordered on basis of the human reference genome, while the vertebrate genomes were ranked in a dendrogram based on GC similarities (Fig. 3a). This visual display captures the overall idea of conservation of GC poor and GC rich regions in vertebrate genomes. The choice of the human reference genome is not determining for these observations. This is shown by using spotted gar (*Lepisosteus oculatus)* as a reference genome for ordering the common vertebrate genes (Fig. 3b and Additional file 7: Figure S6). Using this reference for the ordered list of genes, we still observe the similarities of landscapes when performing intra-mammalian or intra-reptilian comparisons and contrasts between mammalian and reptilian landscapes, analogous as when the genes are displayed on the human genome order (Fig. 3a versus 3b). The obvious change is the location of peaks and valleys on the ordered set of spotted gar chromosomes.

As the similarities and differences between the GC landscapes follow phylogenetic relationships, we next performed a more systematic analysis based on the calculated integrated geometric distance between landscapes of 55 different vertebrate species. A phylogenetic tree was constructed based on the correlation between the GC content of the genes in 1485 pairwise comparisons. As can be seen in the dendrogram of Fig. 3 and the tanglegram of Additional file 8: Figure S7, with this approach, it was possible to classify all species in their correct taxonomic classes and the geometric distances between species approximate the phylogenetic tree of life.

### The genome-wide GC landscapes of vertebrate mRNAs predict codon usage and the relative rate of protein divergence

**Fig. 4:**
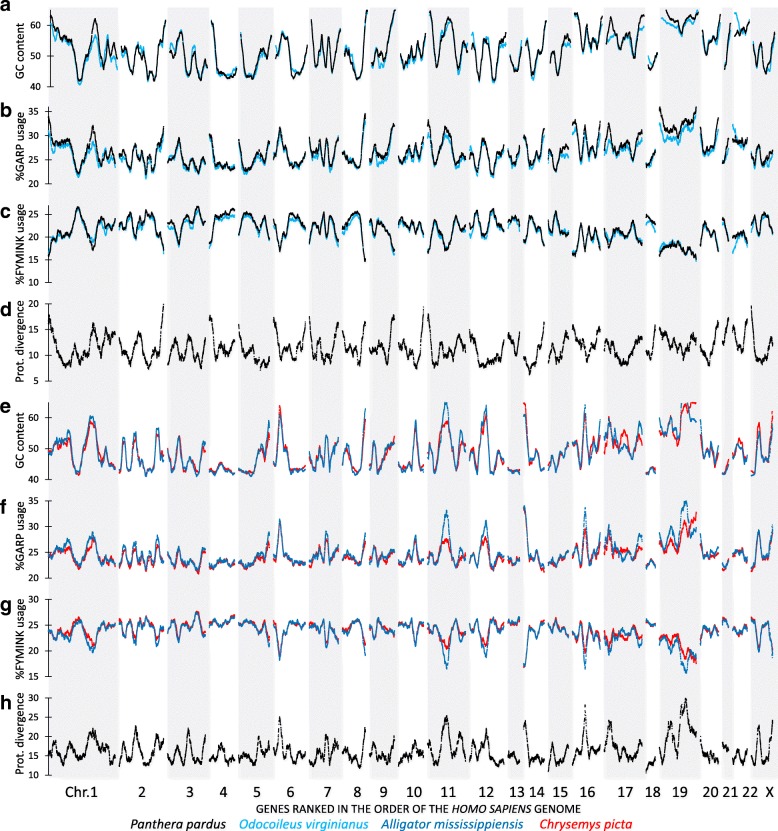
Landscapes of GC content, amino acid usage and protein evolution in mammals and reptiles. Genome-wide landscapes of base composition of predicted mRNAs correlate to the use of amino acids encoded by GC rich and GC depleted codons and to the rate of divergence of orthologous proteins. panels **a**-**d** data for two placental mammals: *Odocoileus virginianus texanus* (white-tailed deer, light blue line) and *Panthera pardus* (leopard, black line). panels **e**-**h** data for non-avian reptiles: *Alligator mississippiensis* (American alligator, dark blue line) and *Chrysemys picta* (painted turtle, red line). GARP% represents the sum of mole % of glycine, alanine, arginine and proline in the amino acid composition of the encoded proteins while FYMINK% represents the sum of mole % of phenylalanine, tyrosine, methionine, isoleucine, asparagine and lysine. The maxima and minima in the landscapes of protein divergence (100% minus protein identity) for the comparison between the two mammals (panel **d**) and the two reptiles (panel **h**) are strongly correlated to the landscapes of amino acid usage (R = 0.92 for GARP and R = -0.95 for FYMINK)

Early studies in unicellular organisms have demonstrated that the genomic base composition has a stochastic influence on the type of codon used for the encoded proteins [21, 22]. A strong effect was seen at the level of the cumulated probability to use glycine, alanine, arginine and proline (GARP – the four amino acids encoded by GC rich codons) as building blocks: this probability being proportional to the GC content of the mRNA sequence [21, 22]. The present analysis shows that the GC landscapes of two mammals (*Odocoileus virginianus texanus* and *Panthera pardus* - Fig. 4a), or two reptiles (*Alligator mississippiensis* and *Chrysemys picta* - Fig. 4e) changes in phase with the corresponding landscapes of GARP content of encoded proteins (Fig. 4b and f). Indeed, the precise location and amplitudes of all of the major peaks at the level of GC content is accurately recapitulated by similar peaks in the landscape of GARP content of encoded proteins. When the genes were ordered on basis of the human reference genome a nearly perfect positive correlation was observed between GC% and GARP%: R = 0.93 (mammals) and R = 0.92 (reptiles). In addition we observed a close to perfect negative correlation, both in the mammalian and reptilian landscapes, between GC% and the sum of amino acids encoded by AU rich codons (phenylalanine, tyrosine, methionine, isoleucine, asparagine and lysine – FYMINK% in Fig. 4c and g) (R = − 0.95 for both mammals and reptiles). For the other 10 amino acids, encoded by “mixed codons” (neither AU-nor GC-rich), a much weaker correlation (− 0.17 < R < − 0.36) with the GC landscape was noted. Correlation plots of amino acid usage and GC content (with and without sliding window) and the landscape data for amino acid usage for the human data are given in Additional file 9: Figure S8.

Together, the analysis of the coding region of the common vertebrate gene set reveals a near perfect positive correlation between GC% and GARP% and near perfect negative correlation with FYMINK%. But the analysis does not show which amino acid substitutions have taken place and which time-dependent succession of changes occurred in each species. We anticipated that such details could enormously vary from species to species, so that the landscapes of divergence rates between pairwise orthologous protein comparisons would be similar to the GC% landscapes. In other words, we would expect accelerated protein evolution in landscape regions of increased GC%. As can be illustrated by the comparison of two mammalian (Fig. 4d) and two reptilian species (Fig. 4h), our prediction was confirmed by the experimental data. For instance, while the average protein divergence rates between *Odocoileus* and *Panthera* was 11% (Fig. 4d), peak levels of protein divergence accelerated to 18%. These peaks coincided exactly with the most prominent peaks of GC content, GARP% and deepest valleys of FYMINK%. With the intra-reptilian pair, *Alligator mississippiensis* versus *Chrysemys picta* we obtained the same overall conclusion (accelerated protein evolution in landscape regions of increased GC%) with the difference that the position of the most prominent peaks and valleys were reptilian-type (Fig. 4h). Again, we excluded that the ordering of the genes on the human reference genome was responsible for our results: by reanalyzing the data, ordered on the *Lepisosteus oculatus* genome (Additional file 10: Figure S9), it is clear that the observed similarity of landscapes of protein divergence, amino acid composition and GC% and their phylogenic basis can be reproduced.

## Discussion

With the current study, we present a novel approach in the field of comparative genomics which uses base- and amino acid composition of the coding genomes of today’s vertebrates in order to visualize changes in genome structure over deep evolutionary time. To achieve this goal, we analyzed the base composition of predicted mRNAs, the amino acid composition of predicted proteins and the divergence of orthologous proteins using a large vertebrate protein-encoding gene set common to 55 vertebrate genomes. For the ordered ranking of genes into regional clusters we used two reference genomes, *Homo sapiens* and *Lepisosteus oculatus*, the spotted gar. The focus of this work was to better visualize powerful effects of deep evolutionary time on the regional base composition of predicted mRNAs and amino acid composition of predicted proteins. We demonstrate with data sets from different mammalian and reptilian species that base composition correlates strongly to the use of amino acids encoded by GC-rich and AU-rich codons. Such correlation becomes much stronger (Additional file 2: Figure S1 and Additional file 8: Figure S7) when we assessed regional context of a gene by averaging the information with its 100 neighbors (50 to each side) to obtain one window signal. When such window signal was allowed to slide over the ordered gene set of the reference genome, the measured parameter followed a landscape with peaks and valleys that was characteristic for the lineage in the phylogenetic tree. During the optimization phase, performed on the human reference gene set, we noted that a larger window size diminished the amplitude of peaks and valleys, while a too small window was more strongly influenced by the erratic effect of widely fluctuating values of neighboring genes (Additional file 2: Figure S1). As suggested by the heatmap of GC content of the 55 vertebrate species (Fig. 3), the genome wide information of GC content of predicted mRNA provides phylogenetic signals that approximate the currently accepted tree of life that is based on DNA sequence information (Additional file 9: Figure S8).

Strong points of our approach are robustness of the data and generalization of the approach. For the current comparative analysis of major vertebrate phyla with evolutionary distances spanning a hundred of millions years or more, the sliding window approach seems a robust tool to visualize strong regional effects of syntenic gene clusters. Base composition outliers (either biologically determined or caused by sequencing artifacts or data base errors) introduce a deviation of less than 1% on the 100 neighbors-averaged data, while the regional fluctuations in the sliding window values amount to 10% or more above or below baseline (Additional file 2: Figure S1). Another strong point of this method is that it can be applied to other sequenced vertebrates, without the need for a physical map. The specific study of teleost genomes which have many extra genes generated by a teleost-specific gene duplication event [23] seems an example of an interesting extension beyond the scope of the present work, but it would require an adaptation of the analyzed gene set and reference genomes.

A first limitation is a potential bias towards vertebrate genomes that are available in public databases such as NCBI, mammals being more represented than amphibians and cartilaginous fish. Because of the teleost-specific gene duplication event [23] too many ambiguities exist in the formation of the orthologous gene pairs with the other vertebrates. Therefore, we excluded information from teleost genomes from the present study. A second limitation is the focus on predicted mRNA sequence, excluding the evolutionary changes in base composition of non-coding and intergenic DNA as well as introns. For the human genome, we assessed inclusion of intron sequence in the sliding window landscapes, expanding the total length of evaluated DNA by one order of magnitude. The data (Additional file 3: Figure S2) clearly show that the position of peaks and valleys in the landscapes remained. Moreover, we confirmed the existence of a higher GC content in exon sequence than in introns [26]. Third, we limited our analysis to what we defined a common vertebrate protein-encoding gene set, excluding lineage-specific genes often with many paralogs which are typically clustered [27]. To assess a potential bias by this selection, we recalculated the GC% landscape for human protein encoding genes with or without the extra set that were not part of the common vertebrate set (difference approximately 4000, mostly mammalian-specific). As can be seen in Additional file 4: Figure S3 limiting our analysis genes to the common vertebrate set did not alter the major characteristics of the GC landscape of the human genes, although details some regions, like chromosome 19 were influenced by extended mammalian-specific gene clusters. The advantage of a common vertebrate gene set was that this approach allowed comparative analysis of reptilian and mammalian landscapes in the same study. One could propose as limitation that we did not dispose over a (complete) physical map of the 55 studied genomes, so that we could not study landscapes of all species using the validated correct gene order of that specific species. However, the phylogenetic relationships of landscapes (intra-mammalian and intra-reptilian) remain when changing from the human to the spotted gar reference genome, two species that are separated approximately 430 million years of time from the last common vertebrate ancestor (Fig. 3 and Additional file 7: Figure S6) [28]. Wondering how this strong regional effect of base composition of vertebrate genes is possible, one could propose that over deep evolutionary time, many of the studied vertebrate genes may have been allowed to travel through genome space by chromosomal rearrangements (inversions, translocations, fusions, fissions), but at the same time avoided crossing the borders of the base composition landscape by adhering to their established low or high GC environment. When linked to the concept of isochores, it seems conceivable that the shuffling of isochores but not the thorough mixing of isochores is the preferred modality of change during genome evolution.

The mechanism responsible for an accumulation of guanine and cytosine bases in particular chromosomal locations during deep evolutionary time has been described and termed GC biased gene conversion (gBGC) [5, 8, 11]. During meiotic recombination, mismatch ambiguities are resolved with a slightly higher proportion of repair in favor of the G or C containing allele. gBGC is considered the driving force for enhancing GC content in particular genomic regions during a long evolution period. When such regions are rearranged out of the active zone of recombination, GC accumulation stops; when on the contrary new regions are reshuffled into the active area of recombination, a new region of GC accumulation starts to form. Peaks in the GC content landscape that are shared by reptiles and mammals (on the human genome order) are most likely phylogenetically old areas of GC biased gene conversion and are located in the chromosome interior on the human reference genome, whereas the typical mammalian specific maxima of GC content occur towards the end of the chromosomes. Thus, while gBGC targets genes located at the end of the chromosomes, the evolutionary rearrangements by chromosome fusion and breakage can remove gene regions away from the subtelomeres and stop GC accumulation and the accelerated protein evolution.

The repartition of genome information over chromosomes that recombine in certain regions during sexual reproduction has major consequences at different levels. First, this type of structural organization can form new epistatic effects between different mutations on neighboring genes for the next generation. Over millions of years of time, the consistent recombination in certain regions drives a progressive change of base composition that strongly correlates to changes in the amino acid composition for half of the amino acids (GARP and FYMINK) of the encoded proteins.

One could say that this correlation between base and amino acid composition is trivial given the nature of the genetic code. But the very nature of a shift from FYMINK to GARP in regions associated with increased GC content is interesting in itself. It was measured before in bacteria [21] and protists [22] consistent with the universal nature of the genetic code. Interestingly, the enrichment of glycine and especially alanine, the chemically least complex amino acids goes at the expense of the complex building blocks such as phenylalanine and tyrosine. For auxotrophic organisms, glycine and alanine are formed in short metabolic pathways compared to the complicated biosynthesis of aromatic amino acids. For heterotrophs, the shift from FYMINK to GARP diminishes the need for some of the most limiting essential amino acids in diets based on plants. When extrapolated to an even earlier time point a change from the use of FYMINK to GARP could represent the recapitulation of a more primitive code that evolved before the origin of the first cells [29]. Looking at the genetic code of today, we notice that that for all of the 16 GC-rich GARP codons the first two bases discriminate, while the third codon base is always redundant. On the contrary, for the 16 AU-rich codons encoding FYMINK, the third code is highly discriminating, including the start and two of the three stop codons. It seems to make sense that regions with gBGC-driven GC accumulation and accelerated mutagenesis have a bias for codons that are fourfold degenerate [30] and more robust in terms of avoiding nonsense mutations [31]. These ideas do not exclude that other effects of the FYMINK to GARP shift exist. One possibility is an impact on protein structure, such as the propensity to be part of alpha helical, coiled coil, beta sheets or collagen helix structures.

## Conclusion

In conclusion, our study of genome-wide GC content landscapes presents a new approach in which genome-wide structural influences on base- and amino acid composition of vertebrate genomes can be studied over deep evolutionary time.

Based on a vertebrate core gene set we were able to compare mRNA base characteristics, amino acid composition of the predicted proteins and protein evolution between species. Our method allows graphical representation of this information in, allowing a quick comparison of coding information over several species which have diverged over millions of years. The generated landscapes are in agreement with the isochores, but are much more pronounced and easier to compare. Comparing the genome wide GC landscapes of 55 vertebrates shows both a strong conservation of common GC rich and GC poor areas and lineage specific events of GC enrichment. In general, areas with a high amount of GC content have an increased presence of glycine, alanine, arginine and proline in the protein and display a higher protein divergence. Our approach of GC landscapes provides a tool that may aid further studies on genes retained in a syntenic block of a genome for extended periods of time.

## Methods

### Retrieving of genome data for a typical vertebrate gene set

Human gene data (gene name, description, chromosome name, strand, GC content, Refseq mRNA name, gene start, gene end) were imported with Biomart using the Ensembl genes 84 database and the *Homo sapiens* genes (GRCh38.p5) dataset. Non-protein coding genes were filtered out by deleting entries that did not have a Refseq transcript (NR_ or NX_) number. For genes with multiple transcripts, only the entry with the longest transcript was retained. This resulted in a set of 19,971 unique human genes. From all species, genbank files containing transcriptome information were downloaded from ftp://ftp.ncbi.nih.gov/genomes/. For each protein-coding gene, one representative Refseq transcript or predicted transcript was selected and percent GC and transcript length was calculated with EMBOSS infoseq. Scripts were custom written in Bash, Python and R. All scripts can be retrieved from https://github.com/thomasintveld/vertebrate-landscapes-genomics. Organisms were then compared by exact matching of gene names to human genes. When exact matching could not be performed (e.g. LOC numbers for gene name), a further manual matching was performed based on the conservation of neighboring syntenic genes, gene description and sequence of encoded protein. For this study, we analyzed the protein encoding genes from non-teleost fish (*n* = 3), amphibia (*n* = 2), non-avian reptiles (*n* = 13), avian reptiles (birds, *n* = 16) and mammals (*n* = 21, incl. human), which are all shown in Additional file 1: Table S1. In our analysis we included genes which are at least two times present in fish, amphibia and non-avian reptiles (criteria 2/18) obtaining a set of typical vertebrate genes (15,824 genes).

### Calculation of protein divergence and amino acid usage

Protein identity between pairs of orthologous proteins was calculated using EMBOSS Stretcher (BLOSUM62 substitution matrix). The protein identity score was calculated as the number of matching residues divided by the length minus the number of gaps to exclude low protein identities as a consequence of incomplete sequence information. Orthologue proteins of leopard *(Panthera pardus)*, white-tailed deer (*Odocoileus virginianus texanus*), American alligator (*Alligator mississipiensis*) and painted turtle (*Chrysemys picta*) were compared among each other. Protein identities below 30% were discarded. Protein divergence was calculated as 1 – protein identity.

For each mRNA transcript, the predicted protein sequence was downloaded and the amount of each amino acid was calculated and divided by the total protein length. We distinguished three different groups: amino acids encoded by GC rich codons (glycine, alanine, arginine and proline; known as GARP), amino acids encoded by AU rich codons (phenylalanine, tyrosine, methionine, isoleucine, asparagine and lysine; known as FYMINK) and the mixed amino acids (leucine, serine, cysteine, histidine, glutamine, threonine, valine, aspartate and glutamate).

### Sliding window analysis

For the vertebrate protein-encoding set of 15,824 genes, key metrics (base composition of predicted mRNA, amino acid composition of predicted proteins and protein divergence) were calculated per gene and subsequently coarse-grained using a sliding window. This approach was chosen because it smoothens out local noise and adds regional context. Windows of 100 neighboring genes were defined, sliding one gene location in the reference frame at a time. For every gene on location *L*_*k*_ a sliding window metric was defined averaging the metric’s value over 100 neighboring genes at positions *L*_*k-50*_ to *L*_*k + 50*_.

### Phylogenetic trees

Pairwise pearson correlation between GC content graphs of organisms was calculated in R. A distance matrix was created based on Euclidean distance. A dendrogram was made by hierarchical clustering of the distance matrix. For the standard (currently accepted) phylogenetic tree, data were obtained from timetree.org [28] and also a dendrogram was created. *Python bivittatus* was not present in Timetree.org; therefore the subgenus *Python molurus* was substituted. Both dendrograms were then displayed in a tanglegram with the dendextend package [32].

## Additional files

Additional file 1: **Table S1.** List of the 55 used species in the analysis. Three fish, two amphibians, 13 non-avian reptiles, 16 birds (avian reptiles) and 21 mammals were used in our analysis. The GC content landscapes in the article from the species above listed are marked in blue. (PDF 471 kb)

Additional file 2: **Figure S1.** Effect of increasing sliding window size on landscape of mRNA GC content. In panels A-E the gray dots represent the GC content of human mRNAs for the individual 15,824 vertebrate genes of the study. Superimposed in blue are the landscapes of averaged GC content calculated with a sliding window of a centered gene and its 10 (**panel A**), 50 (**panel B**), 100 (**panel C**), 150 (**panel D**), and 200 (**panel E**) neighboring genes. We chose to use a sliding window of 100 neighboring genes as a tradeoff between clear visibility and loss of details. (PDF 8099 kb)

Additional file 3: **Figure S2.** GC content landscapes of coding sequence, transcript, intron and total gene sequences are strongly correlated. Data from human protein encoding genes were analyzed for the 15,824 vertebrate genes of the study using a sliding window of the gene and its 100 neighboring genes. 1% (coding sequence, CDS), 2% (gene), 33% (intron) and 34% (total genes) of the total amount of the human genome bases are shown. (PDF 3164 kb)

Additional file 4: **Figure S3.** Influence of 4147 extra human protein encoding genes landscape of GC content. Panel **A** shows the human data for the 15,824 vertebrate genes of the study; **panel B** shows the GC landscape including 4147 genes (many clustered olfactory receptor genes and zinc finger genes) that were not included in the vertebrate gene set. Data were analyzed using a sliding window of the gene and its 100 neighboring genes. (PDF 1946 kb)

Additional file 5: **Figure S4.** Comparison of human GC content landscapes based on numerical order and on physical position. Panel **A** shows the human data for the 15,824 vertebrate genes of the study, each gene being positioned on a linear distance scale of the human chromosomes; **panel B** shows the GC landscape on basis of numerical ranking of the same genes in the human genome. Data were analyzed using a sliding window of the gene and its 100 neighboring genes. (PDF 1751 kb)

Additional file 6: **Figure S5.** Pairwise comparisons of landscapes of genome-wide GC content mRNAs from a mammalian and reptilian species. Panel **A** *Loxodonta africana* (African elephant) versus *Pogona vitticeps* (bearded dragon), **Panel B** *Rhinolophus sinicus* (Chinese horseshoe bat) versus *Aquila chrysaetos canadensis* (American golden eagle), **Panel C** *Physeter catodon* (sperm whale) versus *Chrysemys picta* (painted turtle). Genes were ranked on the order of the human genome and GC content was calculated using a sliding window approach of a gene and its 100 surrounding genes. The three species from each clade have a similar GC content profile, but major differences can be seen between the two clades. There are no clear differences observed for species which live on land (**A**), can fly (**B**) and swim (**C**). (PDF 4372 kb)

Additional file 7: **Figure S6.** mRNA GC content landscapes from four mammals and four reptiles ranked in a non-human genome. Genes of the same species as in Figs. 1 and 2 of the study were ranked on the order of the *Lepisosteus oculatus* (spotted gar) genome and GC content was calculated with the sliding window approach. (PDF 5111 kb)

Additional file 8: **Figure S7.** Comparison of phylogenetic trees of 55 vertebrate species. The phylogenetic signal on the left side was assembled based on the correlation between the GC content of genes. The tree on the right side was assembled based on Time Tree of Life [28]. The relationship between both phylogenetic trees is displayed in a tanglegram. (PDF 45 kb)

Additional file 9: **Figure S8.** Correlation between GC content of mRNA and GARP% and FYMINK% in encoded proteins. The human data for the 15,824 vertebrate genes were assessed at the level of sliding window 100 means (**panels A-C**) or values for individual genes of the study (**panels A’-C′**). In both cases GARP% was positively and FYMINK% negatively correlated to GC content, while the abundance of the other ten amino acids was not influenced by GC content. **Panel D** shows perfectly mirrored landscapes of GARP% (red; genome-wide average = 26.1%) and FYMINK% (blue; genome-wide average = 22.4%), while the sum of the other ten amino acids (black; genome-wide average = 51.5% of amino acid composition) is not dependent on genomic position. (PDF 7706 kb)

Additional file 10: **Figure S9.** Landscapes of mammals and reptiles, ranked on the spotted gar genome order. Same data as in Fig. 4, but the genes are now ranked on the order of *Lepisosteus oculatus* (spotted gar) genome. The fluctuations of GC content, amino acid usage and protein divergence correlate as depicted in Fig. 4. R-values for GC content: between *Panthera pardus* and *Odocoileus virginianus texanus* (R = 0.97); between *Alligator mississippiensis* and *Chrysemys picta* (R = 0.96). (XLSX 14581 kb) (PDF 8947 kb)

Additional file 11: **Table S2.** Transcript ID and GC content of all 55 used species. (XLSX 14581 kb)

## Data Availability

Data were extracted from publicly available genbank files. Further datasets generated and/or analyzed during the current study are available from the corresponding author on reasonable request. A list of the 15,824 used genes, NM/XM-numbers and GC content for all species used in our analysis can be found in Additional file 11.

## Abbreviations

FYMINK%: Sum of the molar percentage phenylalanine (F), tyrosine (Y), methionine (M), isoleucine (I), asparagine (N) and lysine (K) in the amino acid composition of the encoded protein.
GARP%: Sum of the molar percentage of glycine (G), alanine (A), arginine (R) and proline (P) in the amino acid composition of the encoded protein.
gBGC: GC biased gene conversion
