# Supplementary material for: GC content of vertebrate exome landscapes reveal areas of accelerated protein evolution
Source: BMC Evol Biol. 2019 Jul 16;19:144. doi: 10.1186/s12862-019-1469-1 (PMC6636035; doi:10.1186/s12862-019-1469-1)
Supplement: Supplementary file 1 — Table S1. List of the 55 used species in the analysis. Three fish, two amphibians, 13 non-avian reptiles, 16 birds (avian reptiles) and 21 mammals were used in our analysis. The GC content landscapes in the article from the species above listed are marked in blue. (PDF 471 kb) [file 12862_2019_1469_MOESM1_ESM.pdf]

Additional file 1: Table S1: List of the 55 used species in the analysis

	English name	*Latin name*	*References*
FISH (n=3)	Elephant shark	*Callorhinchus milii*	[1]
	Coelacanth	*Latimeria chalumnae*	[2]
	Spotted gar	*Lepisosteus oculatus*	[3]
AMPHIBIA (n=2)	Tibetian frog	*Nanorana parkeri*	[4]
	Tropical clawed frog	*Xenopus tropicalis*	[5]
NON-AVIAN REPTILES (n=13)	American alligator	*Alligator mississippiensis*	[6]
	Chinese alligator	*Alligator sinensis*	[7]
	Green anole	*Anolis carolinensis*	[8]
	Green sea turtle	*Chelonia mydas*	[9]
	Painted turtle	*Chrysemys picta*	[10]
	Australian saltwater crocodile	*Crocodylus porosus*	[6]
	Gharial	*Gavialis gangeticus*	[6]
	Schlegel's Japanese Gecko	*Gekko japonicus*	[11]
	Chinese soft-shelled turtle	*Pelodiscus sinensis*	[9]
	Central bearded dragon	*Pogona vitticeps*	[12]
	Brown-spotted pit viper	*Protobothrops mucrosquamatus*	[13]
	Burmese python	*Python bivittatus*	[14]
	Common garter snake	*Thamnophis sirtalis*	
AVIAN REPTILES (BIRDS) (n=16)	Mallard	*Anas platyrhynchos*	[15]
	Domestic goose	*Anser cygnoides domesticus*	[16]
	Emperor penguin	*Aptenodytes forsteri*	[17]
	Brown kiwi	*Apteryx australis mantelli*	[18]
	American golden eagle	*Aquila chrysaetos canadensis*	[19]
	Anna's hummingbird	*Calypte anna*	[17]
	Rock pigeon	*Columba livia*	[17]
	American crow	*Corvus brachyrhynchos*	[17]
	Japanese quail	*Coturnix japonica*	[20]
	Chicken	*Gallus gallus*	[17,21,22]
	Bengalese finch	*Lonchura striata domestica*	[23]
	Great Tit	*Parus major*	[24]
	Tibetan ground-tit	*Pseudopodoces humilis*	[25]
	Ostrich	*Struthio camelus australis*	[17]
	Common starling	*Sturnus vulgaris*	[26]
	White-throated tinamou	*Tinamus guttatus*	[17]
MAMMALS (n=21)	Giant panda	*Ailuropoda melanoleuca*	[27]
	Western gorilla	*Gorilla gorilla*	[28,29]
	Great roundleaf bat	*Hipposideros armiger*	[30]
	Human	*Homo sapiens*	[31,32]
	African savanna elephant	*Loxodonta africana*	[33]
	Natal long-fingered bat	*Miniopterus natalensis*	[34]
	Gray short-tailed opossum	*Monodelphis domestica*	[35]
	House mouse	*Mus musculus*	[36]
	Brandt's bat	*Myotis brandtii*	[37]
	David's Myotis	*Myotis davidii*	[38]
	Little brown bat	*Myotis lucifugus*	[33]
	White-tailed deer	*Odocoileus virginianus texanus*	[39]
	Leopard	*Panthera pardus*	

	Amur tiger	*Panthera tigris altaica*	[40]
	Koala	*Phascolarctos cinereus*	[41]
	Sperm whale	*Physeter catodon*	
	Large flying fox	*Pteropus vampyrus*	
	Chinese rufous horseshoe bat	*Rhinolophus sinicus*	[30]
	Tasmanian devil	*Sarcophilus harrisii*	[42]
	Pig	*Sus scrofa*	[43]
	Polar bear	*Ursus maritimus*	[44]

1. Venkatesh B, Lee AP, Ravi V, Maurya AK, Lian MM, Swann JB, et al. Elephant shark genome provides unique insights into gnathostome evolution. Nature. 2014;505:174–9.
2. Amemiya CT, Alföldi J, Lee AP, Fan S, Philippe H, Maccallum I, et al. The African coelacanth genome provides insights into tetrapod evolution. Nature. 2013;496:311–6.
3. Braasch I, Gehrke AR, Smith JJ, Kawasaki K, Manousaki T, Pasquier J, et al. The spotted gar genome illuminates vertebrate evolution and facilitates human-teleost comparisons. Nat. Genet. 2016;48.
4. Sun Y-B, Xiong Z-J, Xiang X-Y, Liu S-P, Zhou W-W, Tu X-L, et al. Whole-genome sequence of the Tibetan frog *Nanorana parkeri* and the comparative evolution of tetrapod genomes. Proc. Natl. Acad. Sci. 2015;112:E1257–62.
5. Session AM, Uno Y, Kwon T, Chapman JA, Toyoda A, Takahashi S, et al. Genome evolution in the allotetraploid frog Xenopus laevis. Nature. Nature Publishing Group; 2016;538:336–43.
6. Green RE, Braun EL, Armstrong J, Earl D, Nguyen N, Hickey G, et al. Three crocodilian genomes reveal ancestral patterns of evolution among archosaurs. Science (80-.). 2014;346:1355-.
7. Wan QH, Pan SK, Hu L, Zhu Y, Xu PW, Xia JQ, et al. Genome analysis and signature discovery for diving and sensory properties of the endangered Chinese alligator. Cell Res. 2013;23:1091–105.
8. Alföldi J, Di Palma F, Grabherr M, Williams C, Kong L, Mauceli E, et al. The genome of the green anole lizard and a comparative analysis with birds and mammals. Nature. 2011;477:587–91.
9. Wang Z, Pascual-Anaya J, Zadissa A, Li W, Niimura Y, Huang Z, et al. The draft genomes of soft-shell turtle and green sea turtle yield insights into the development and evolution of the turtle-specific body plan. Nat. Genet. 2013;45:701–6.
10. Bradley Shaffer H, Minx P, Warren DE, Shedlock AM, Thomson RC, Valenzuela N, et al. The western painted turtle genome, a model for the evolution of extreme physiological adaptations in a slowly evolving lineage. Genome Biol. 2013;14.
11. Liu Y, Zhou Q, Wang Y, Luo L, Yang J, Yang L, et al. Gekko japonicus genome reveals evolution of adhesive toe pads and tail regeneration. Nat. Commun. Nature Publishing Group; 2015;6:1–11.
12. Georges A, Li Q, Lian J, O'Meally D, Deakin J, Wang Z, et al. High-coverage sequencing and annotated assembly of the genome of the Australian dragon lizard Pogona vitticeps. Gigascience. 2015;4.
13. Aird SD, Arora J, Barua A, Qiu L, Terada K, Mikheyev AS. Population genomic analysis of a pitviper reveals microevolutionary forces underlying venom chemistry. Genome Biol. Evol. 2017;9:2640–9.
14. Koning APJ De, Hall KT, Card DC, Drew R, Fujita MK, Ruggiero RP, et al. Correction for Castoe et al., The Burmese python genome reveals the molecular basis for extreme adaptation in snakes. Proc. Natl. Acad. Sci. 2014;111:3194–3194.
15. Huang Y, Li Y, Burt DW, Chen H, Zhang Y, Qian W, et al. The duck genome and transcriptome provide insight into an avian influenza virus reservoir species. Nat. Genet. 2013;45:776–83.
16. Lu L, Chen Y, Wang Z, Li X, Chen W, Tao Z, et al. The goose genome sequence leads to insights into the evolution of waterfowl and susceptibility to fatty liver. Genome Biol. ???; 2015;16:1–11.
17. Zhang G, Li C, Li Q, Li B, Larkin DM, Lee C, et al. Comparative genomics reveals insights into avian genome evolution and adaptation. Science (80-.). 2014;346:1311–20.
18. Le Duc D, Renaud G, Krishnan A, Almén MS, Huynen L, Prohaska SJ, et al. Kiwi genome provides insights into evolution of a nocturnal lifestyle. Genome Biol. Genome Biology; 2015;16:1–15.
19. Doyle JM, Katzner TE, Bloom PH, Ji Y, Wijayawardena BK, DeWoody JA. The genome sequence of a widespread apex predator, the golden eagle (Aquila chrysaetos). PLoS One. 2014;9:20–2.
20. Kawahara-Miki R, Sano S, Nunome M, Shimmura T, Kuwayama T, Takahashi S, et al. Next-generation sequencing reveals genomic features in the Japanese quail. Genomics. Elsevier Inc.; 2013;101:345–53.
21. International Chicken Genome Sequencing Consortium. Sequence and comparative analysis of the chicken genome provide unique perspectives on vertebrate evolution. Nature. 2004;432:695–716.

22. Thomas S, Underwood JG, Tseng E, Holloway AK. Long-read sequencing of chicken transcripts and identification of new transcript isoforms. PLoS One. 2014;9:1–6.
23. Colquitt BM, Mets DG, Brainard MS. Draft genome assembly of the Bengalese finch, Lonchura striata domestica, a model for motor skill variability and learning. Gigascience. 2018;7:1–6.
24. Laine VN, Gossmann TI, Schachtschneider KM, Garroway CJ, Madsen O, Verhoeven KJF, et al. Evolutionary signals of selection on cognition from the great tit genome and methylome. Nat. Commun. 2016;7:1–9.
25. Cai Q, Qian X, Lang Y, Luo Y, Xu J, Pan S, et al. Genome sequence of ground tit Pseudopodoces humilis and its adaptation to high altitude. Genome Biol. 2015;14:R29.
26. Richardson MF, Sherwin WB, Rollins LA. De Novo Assembly of the Liver Transcriptome of the European Starling, Sturnus vulgaris. J. genomics. 2017;5:54–7.
27. Li R, Fan W, Tian G, Zhu H, He L, Cai J, et al. The sequence and de novo assembly of the giant panda genome. Nature. 2010;463:311–7.
28. Gordon D, Huddleston J, Chaisson MJP, Hill CM, Kronenberg ZN, Munson KM, et al. Long-read sequence assembly of the gorilla genome. Science. 2016;352:aae0344.
29. Scally A, Dutheil JY, Hillier LW, Jordan GE, Goodhead I, Herrero J, et al. Insights into hominid evolution from the gorilla genome sequence. Nature. Nature Publishing Group; 2012;483:169–75.
30. Dong D, Lei M, Hua P, Pan YH, Mu S, Zheng G, et al. The genomes of two bat species with long constant frequency echolocation calls. Mol. Biol. Evol. 2017;34:20–34.
31. Lander ES, Linton LM, Birren B, Nusbaum C, Zody MC, Baldwin J, et al. Initial sequencing and analysis of the human genome. Nature. 2001;409:860–921.
32. Venter JC, Adams MDM, Myers EEW, Li PW, Mural RJ, Sutton GG, et al. The sequence of the human genome. Sci. …. 2001;291:1304–52.
33. Lindblad-Toh K, Garber M, Zuk O, Lin MF, Parker BJ, Washietl S, et al. A high-resolution map of human evolutionary constraint using 29 mammals. Nature. Nature Publishing Group; 2011;478:476–82.
34. Eckalbar WL, Schlebusch SA, Mason MK, Gill Z, Parker A V., Booker BM, et al. Transcriptomic and epigenomic characterization of the developing bat wing. Nat. Genet. 2016;48:528–36.
35. Mikkelsen TS, Wakefield MJ, Aken B, Amemiya CT, Chang JL, Duke S, et al. Genome of the marsupial Monodelphis domestica reveals innovation in non-coding sequences. Nature. 2007;447:167–77.
36. Waterston RH, Lindblad-Toh K, Birney E, Rogers J, Abril JF, Agarwal P, et al. Initial sequencing and comparative analysis of the mouse genome. Nature. 2002;420:520–62.
37. Seim I, Fang X, Xiong Z, Lobanov A V., Huang Z, Ma S, et al. Genome analysis reveals insights into physiology and longevity of the Brandt's bat Myotis brandtii. Nat. Commun. Nature Publishing Group; 2013;4:1–8.
38. Zhang G, Cowled C, Shi Z, Huang Z, Bishop-Lilly K a, Fang X, et al. Comparative Analysis of Bat Genomes. Science (80-.). 2013;339:456–60.
39. Sabury CM, Bhattarai EK, Taylor JF, Viswanathan GG, Cooper SM, Davis DS, et al. Genome-wide polymorphism and comparative analyses in the white-tailed deer (Odocoileus virginianus): A model for conservation genomics. PLoS One. 2011;6.
40. Cho YS, Hu L, Hou H, Lee H, Xu J, Kwon S, et al. The tiger genome and comparative analysis with lion and snow leopard genomes. Nat. Commun. 2013;4.
41. Hobbs M, Pavasovic A, King AG, Prentis PJ, Eldridge MDB, Chen Z, et al. A transcriptome resource for the koala (Phascolarctos cinereus): Insights into koala retrovirus transcription and sequence diversity. BMC Genomics. 2014;15:1–16.
42. Murchison EP, Schulz-Trieglaff OB, Ning Z, Alexandrov LB, Bauer MJ, Fu B, et al. Genome sequencing and analysis of the Tasmanian devil and its transmissible cancer. Cell. Elsevier Inc.; 2012;148:780–91.
43. Groenen MAM, Archibald AL, Uenishi H, Tuggle CK, Takeuchi Y, Rothschild MF, et al. Analyses of pig genomes provide insight into porcine demography and evolution. Nature. Nature Publishing Group; 2012;491:393–8.
44. Liu S, Lorenzen ED, Fumagalli M, Li B, Harris K, Xiong Z, et al. Population genomics reveal recent speciation and rapid evolutionary adaptation in polar bears. Cell. 2014;157:785–94.
