# Supplementary figures and images for: GC content of vertebrate exome landscapes reveal areas of accelerated protein evolution
Source: BMC Evol Biol. 2019 Jul 16;19:144. doi: 10.1186/s12862-019-1469-1 (PMC6636035; doi:10.1186/s12862-019-1469-1)

**A**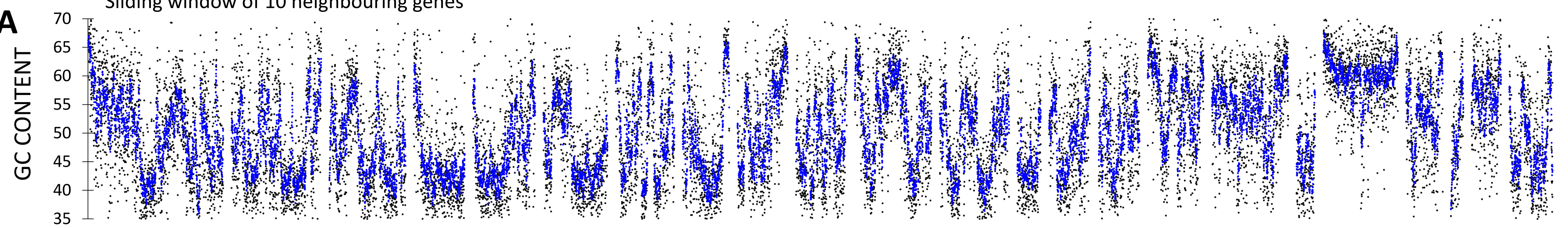**B**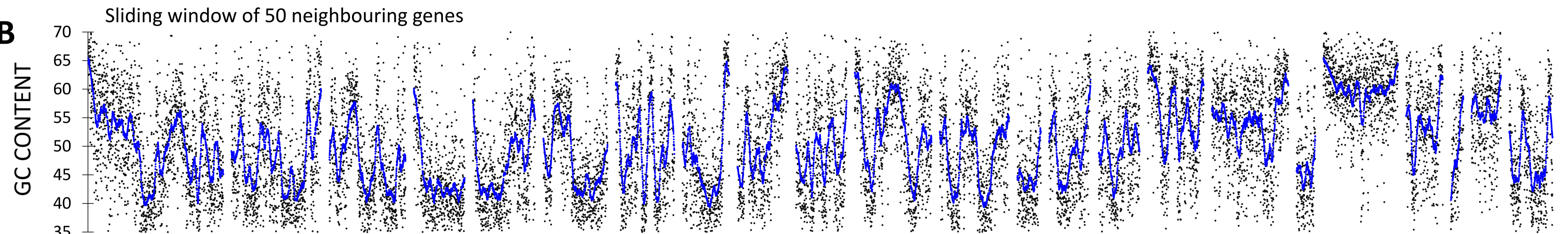**C**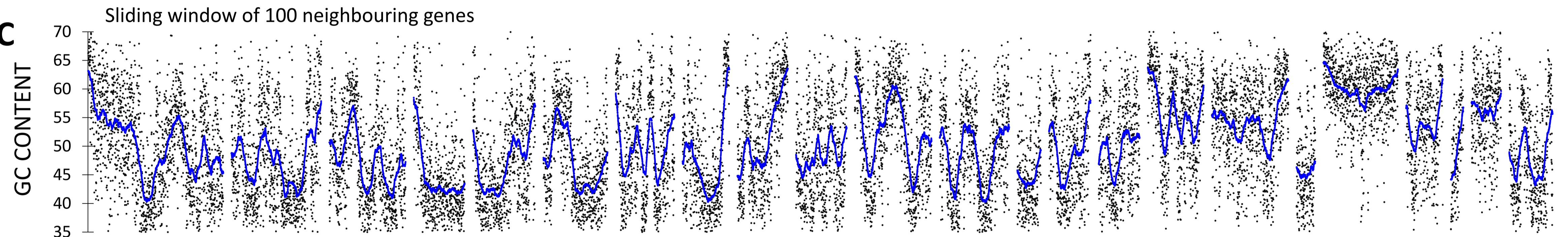**D**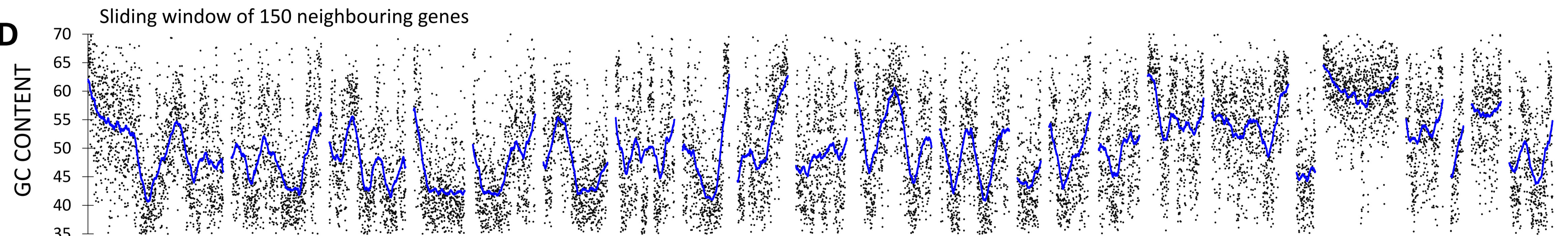**E**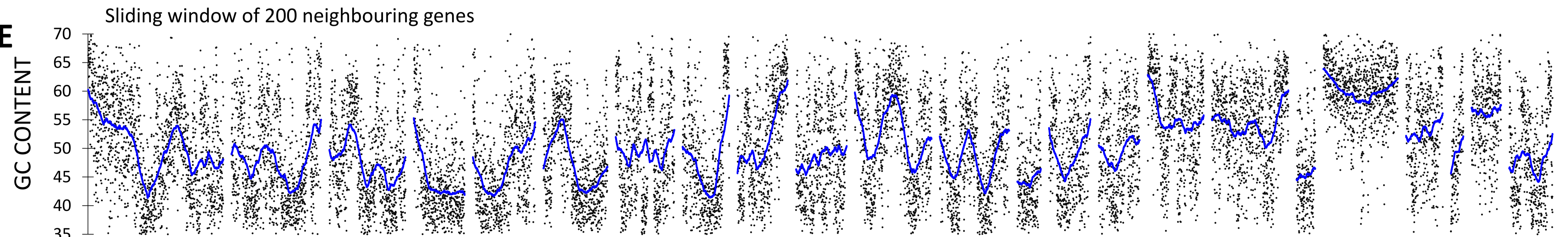

Chr.1

2

3

4

5

6

7

8

9

10

11

12

13

14

15

16

17

18

19

20

21

22

X

GENES RANKED IN THE ORDER OF THE *HOMO SAPIENS* GENOME

Supplement: Supplementary file 2 — Figure S1. Effect of increasing sliding window size on landscape of mRNA GC content. In panels A-E the gray dots represent the GC content of human mRNAs for the individual 15,824 vertebrate genes of the study. Superimposed in blue are the landscapes of averaged GC content calculated with a sliding window of a centered gene and its 10 (panel A), 50 (panel B), 100 (panel C), 150 (panel D), and 200 (panel E) neighboring genes. We chose to use a sliding window of 100 neighboring genes as a tradeoff between clear visibility and loss of details. (PDF 8099 kb) [file 12862_2019_1469_MOESM2_ESM.pdf]

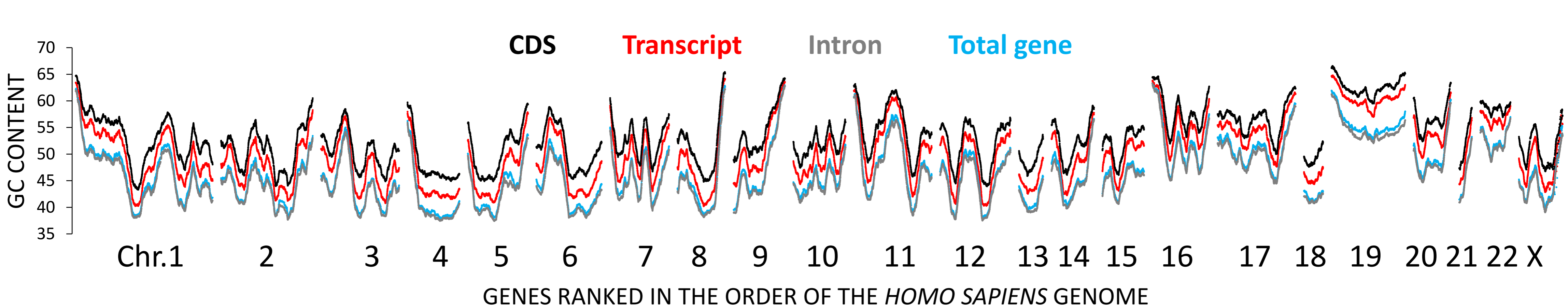

Supplement: Supplementary file 3 — Figure S2. GC content landscapes of coding sequence, transcript, intron and total gene sequences are strongly correlated. Data from human protein encoding genes were analyzed for the 15,824 vertebrate genes of the study using a sliding window of the gene and its 100 neighboring genes. 1% (coding sequence, CDS), 2% (gene), 33% (intron) and 34% (total genes) of the total amount of the human genome bases are shown. (PDF 3164 kb) [file 12862_2019_1469_MOESM3_ESM.pdf]

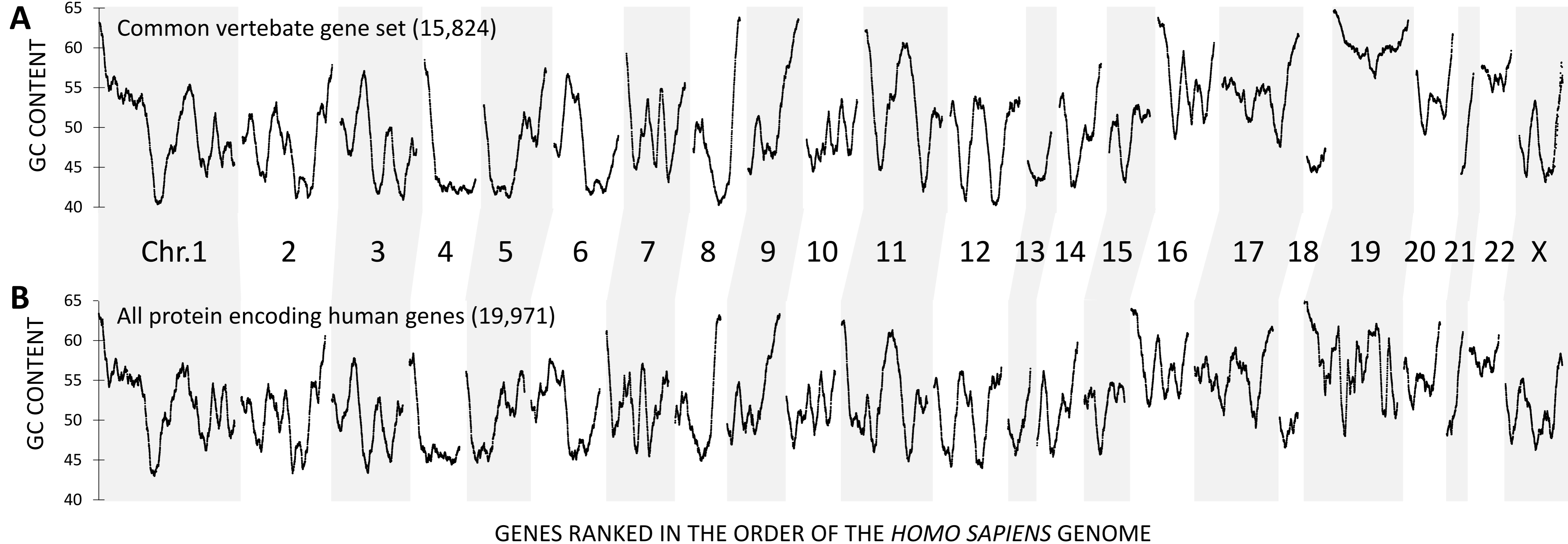

Supplement: Supplementary file 4 — Figure S3. Influence of 4147 extra human protein encoding genes landscape of GC content. Panel A shows the human data for the 15,824 vertebrate genes of the study; panel B shows the GC landscape including 4147 genes (many clustered olfactory receptor genes and zinc finger genes) that were not included in the vertebrate gene set. Data were analyzed using a sliding window of the gene and its 100 neighboring genes. (PDF 1946 kb) [file 12862_2019_1469_MOESM4_ESM.pdf]

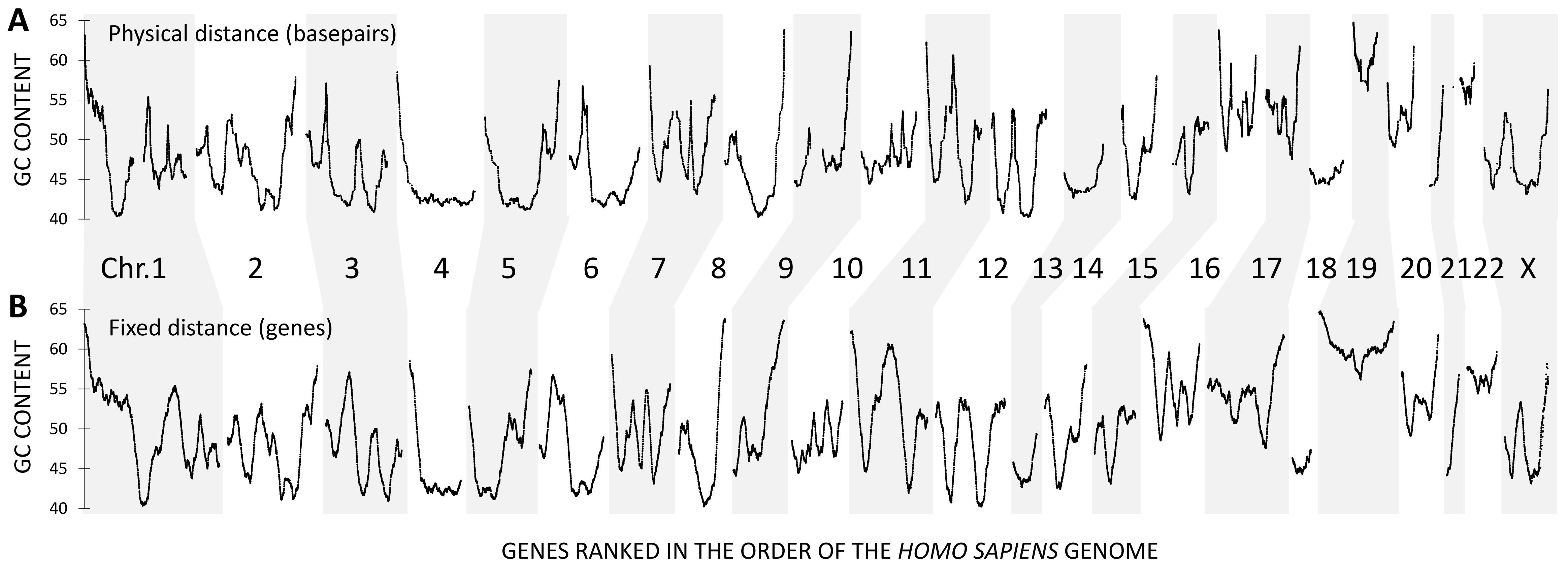

Supplement: Supplementary file 5 — Figure S4. Comparison of human GC content landscapes based on numerical order and on physical position. Panel A shows the human data for the 15,824 vertebrate genes of the study, each gene being positioned on a linear distance scale of the human chromosomes; panel B shows the GC landscape on basis of numerical ranking of the same genes in the human genome. Data were analyzed using a sliding window of the gene and its 100 neighboring genes. (PDF 1751 kb) [file 12862_2019_1469_MOESM5_ESM.pdf]

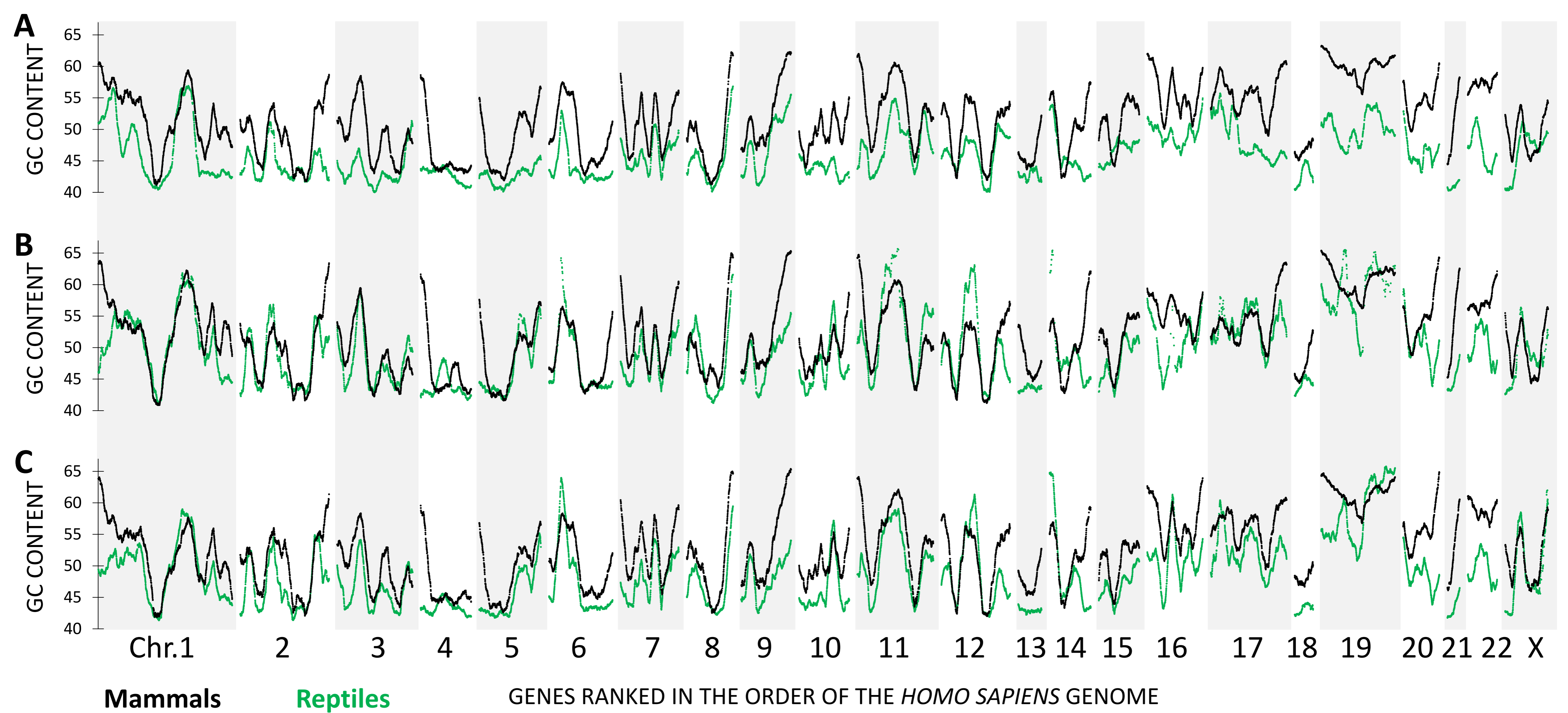

Supplement: Supplementary file 6 — Figure S5. Pairwise comparisons of landscapes of genome-wide GC content mRNAs from a mammalian and reptilian species. Panel A Loxodonta africana (African elephant) versus Pogona vitticeps (bearded dragon), Panel B Rhinolophus sinicus (Chinese horseshoe bat) versus Aquila chrysaetos canadensis (American golden eagle), Panel C Physeter catodon (sperm whale) versus Chrysemys picta (painted turtle). Genes were ranked on the order of the human genome and GC content was calculated using a sliding window approach of a gene and its 100 surrounding genes. The three species from each clade have a similar GC content profile, but major differences can be seen between the two clades. There are no clear differences observed for species which live on land (A), can fly (B) and swim (C). (PDF 4372 kb) [file 12862_2019_1469_MOESM6_ESM.pdf]

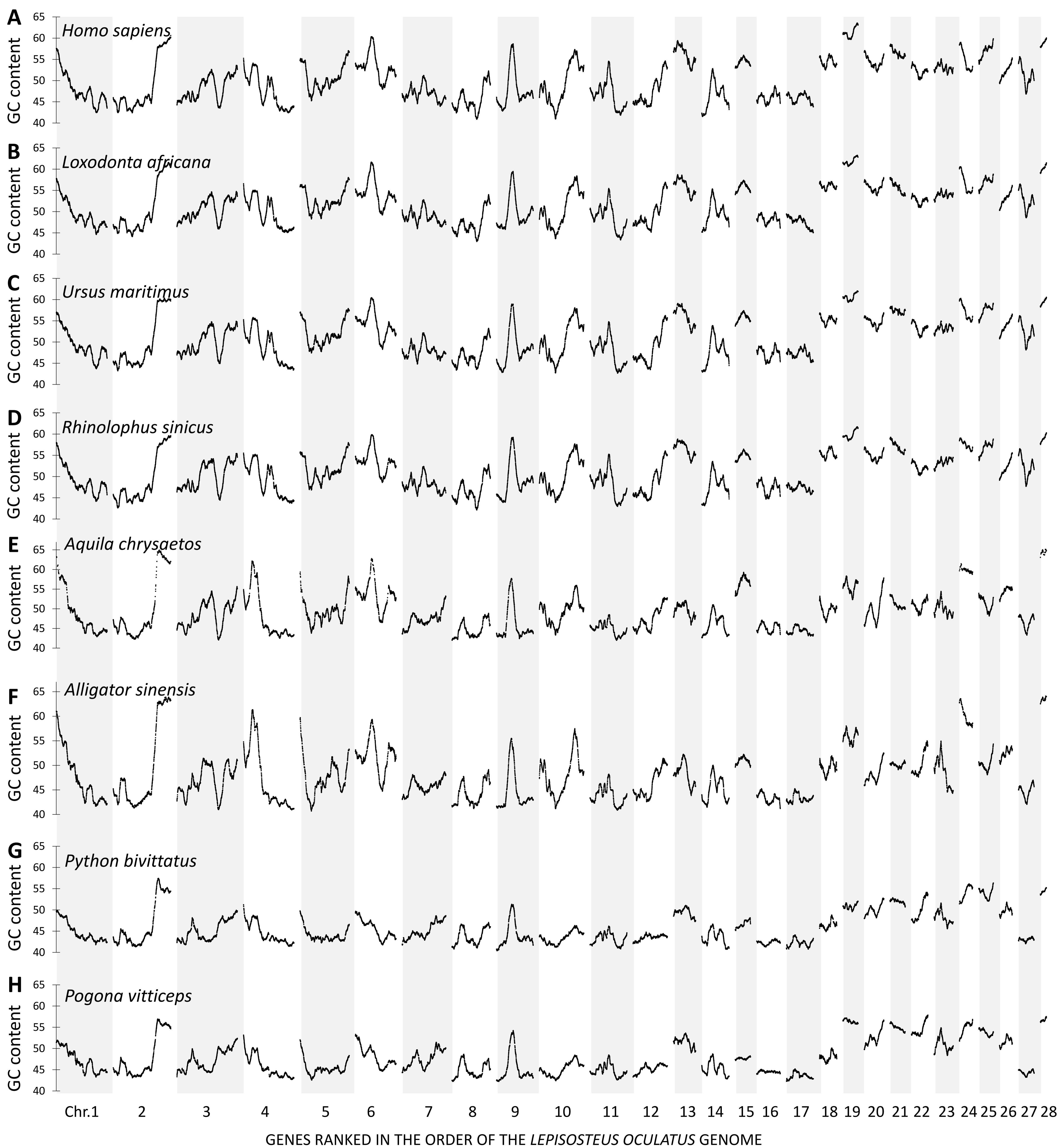

Supplement: Supplementary file 7 — Figure S6. mRNA GC content landscapes from four mammals and four reptiles ranked in a non-human genome. Genes of the same species as in Figs. 1 and 2 of the study were ranked on the order of the Lepisosteus oculatus (spotted gar) genome and GC content was calculated with the sliding window approach. (PDF 5111 kb) [file 12862_2019_1469_MOESM7_ESM.pdf]

entanglement = 0.013

Tree of Life

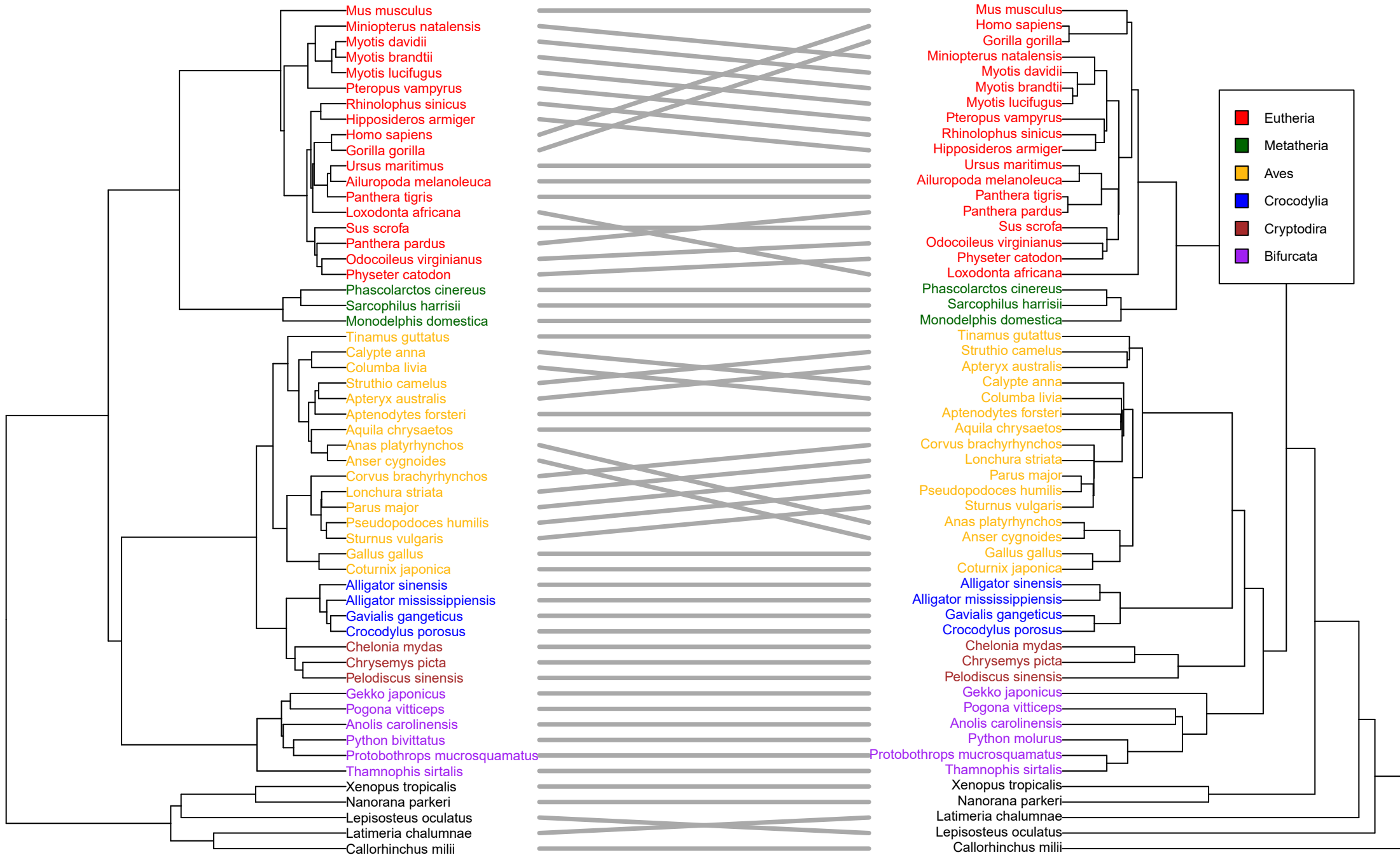

Correlation GC%

0 100 200 300 400

Supplement: Supplementary file 8 — Figure S7. Comparison of phylogenetic trees of 55 vertebrate species. The phylogenetic signal on the left side was assembled based on the correlation between the GC content of genes. The tree on the right side was assembled based on Time Tree of Life [28]. The relationship between both phylogenetic trees is displayed in a tanglegram. (PDF 45 kb) [file 12862_2019_1469_MOESM8_ESM.pdf]

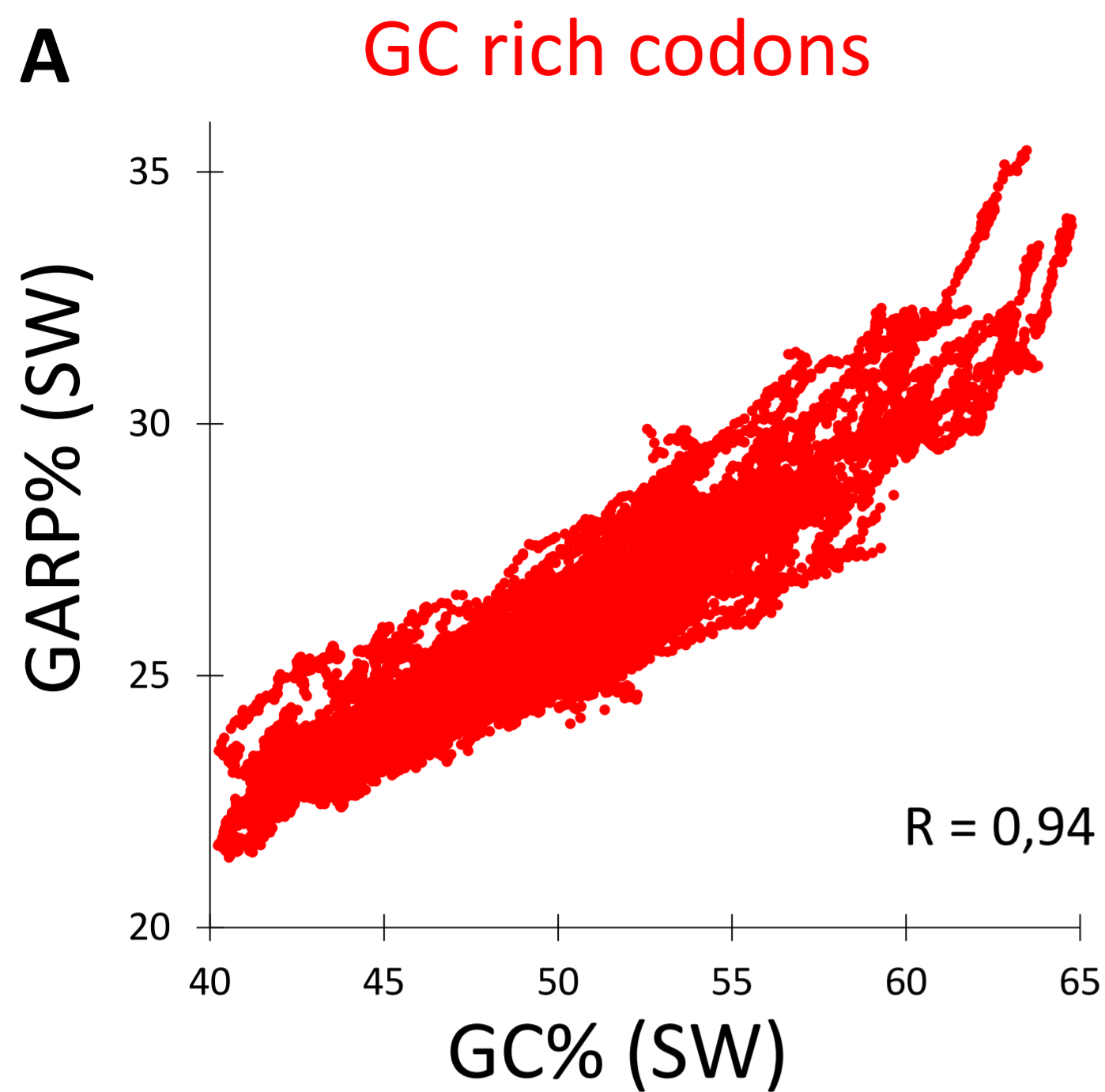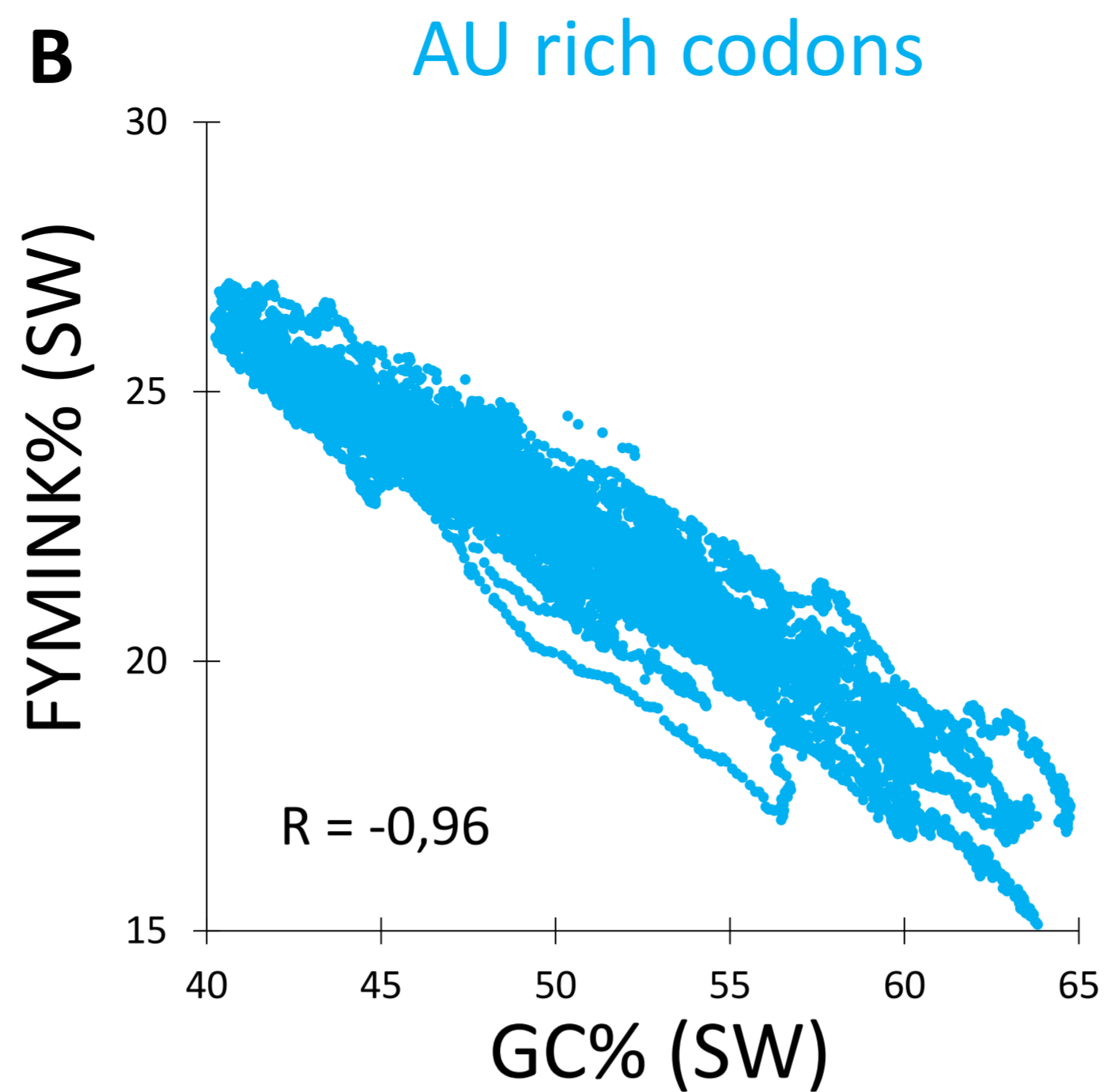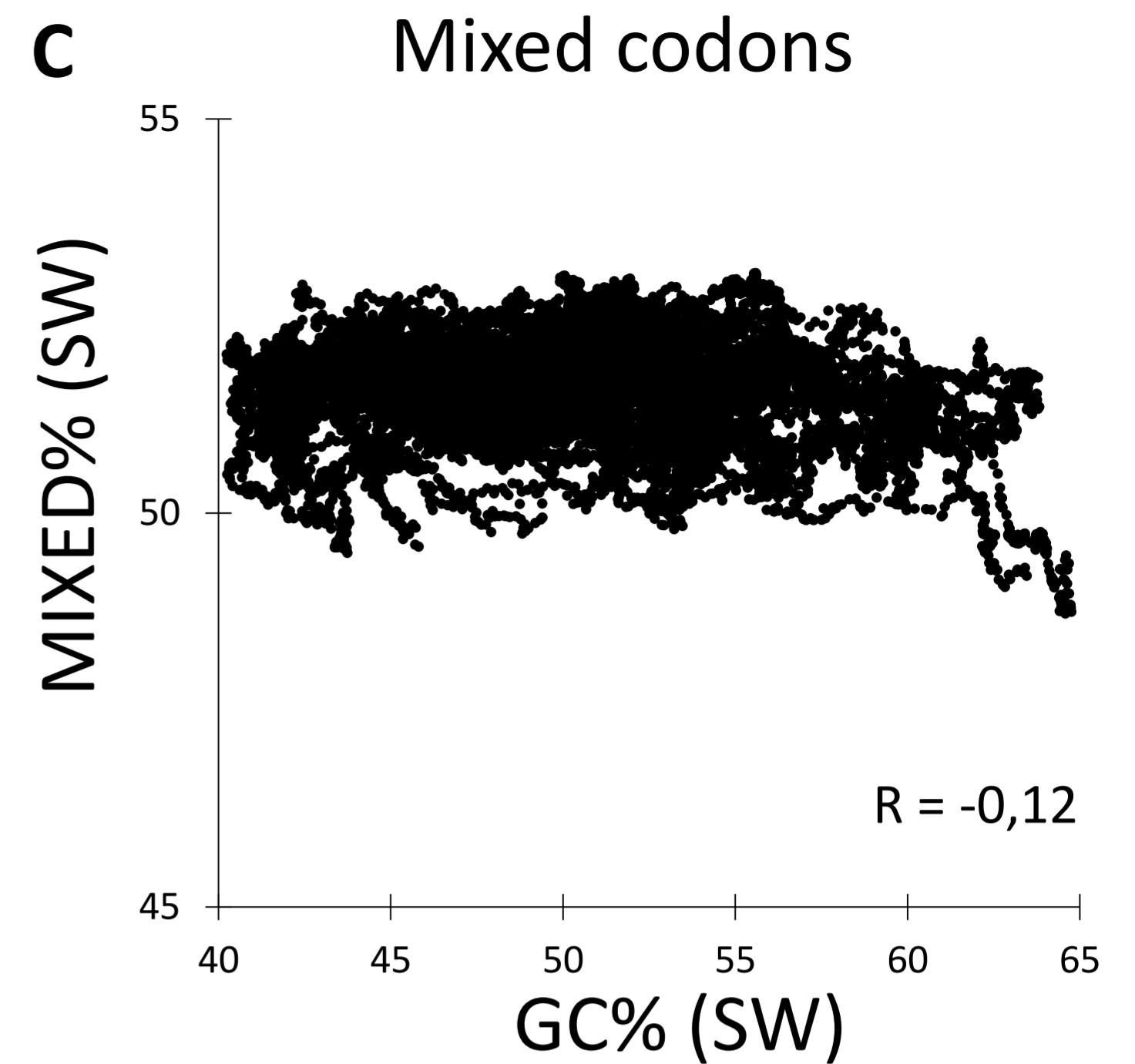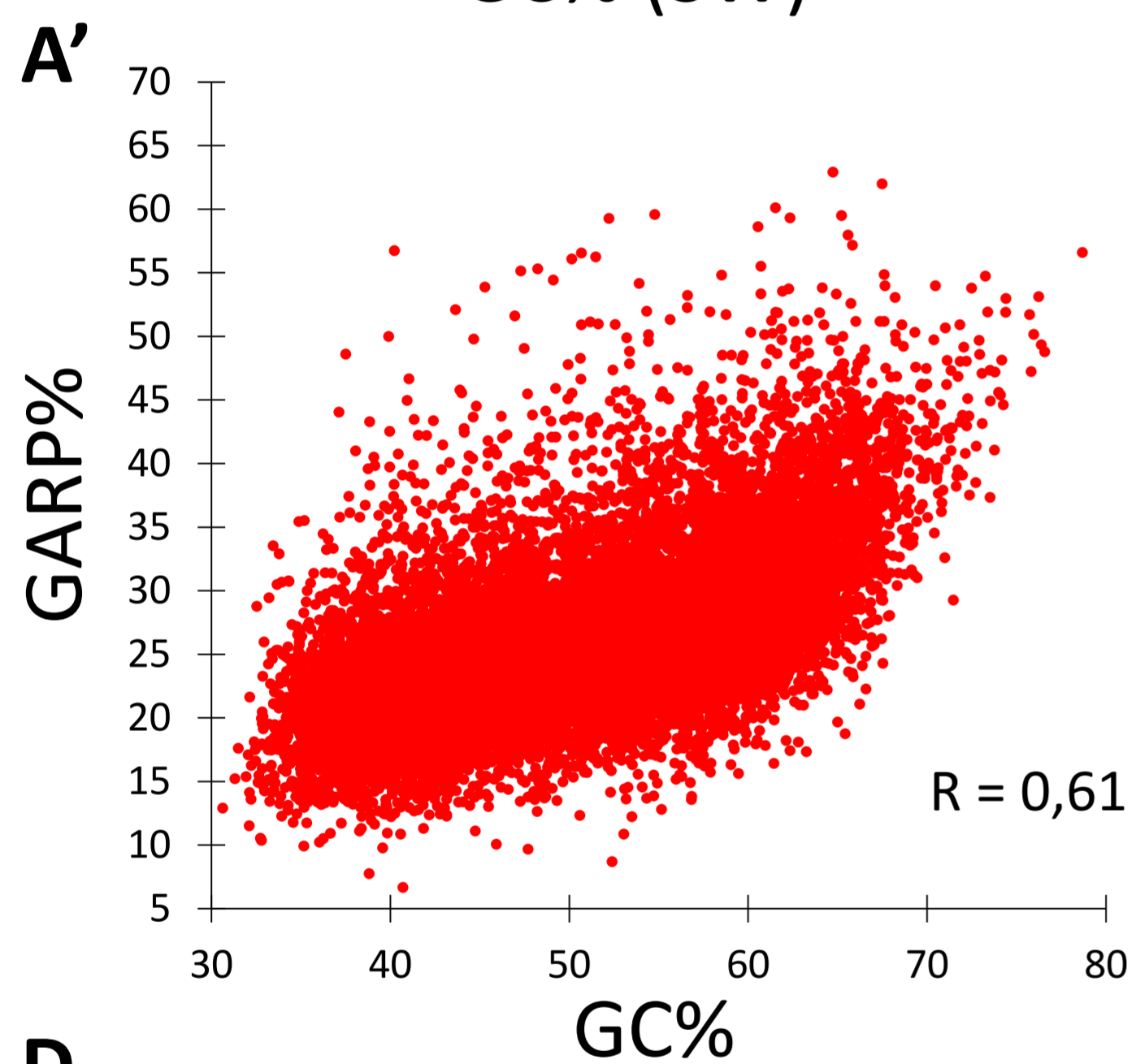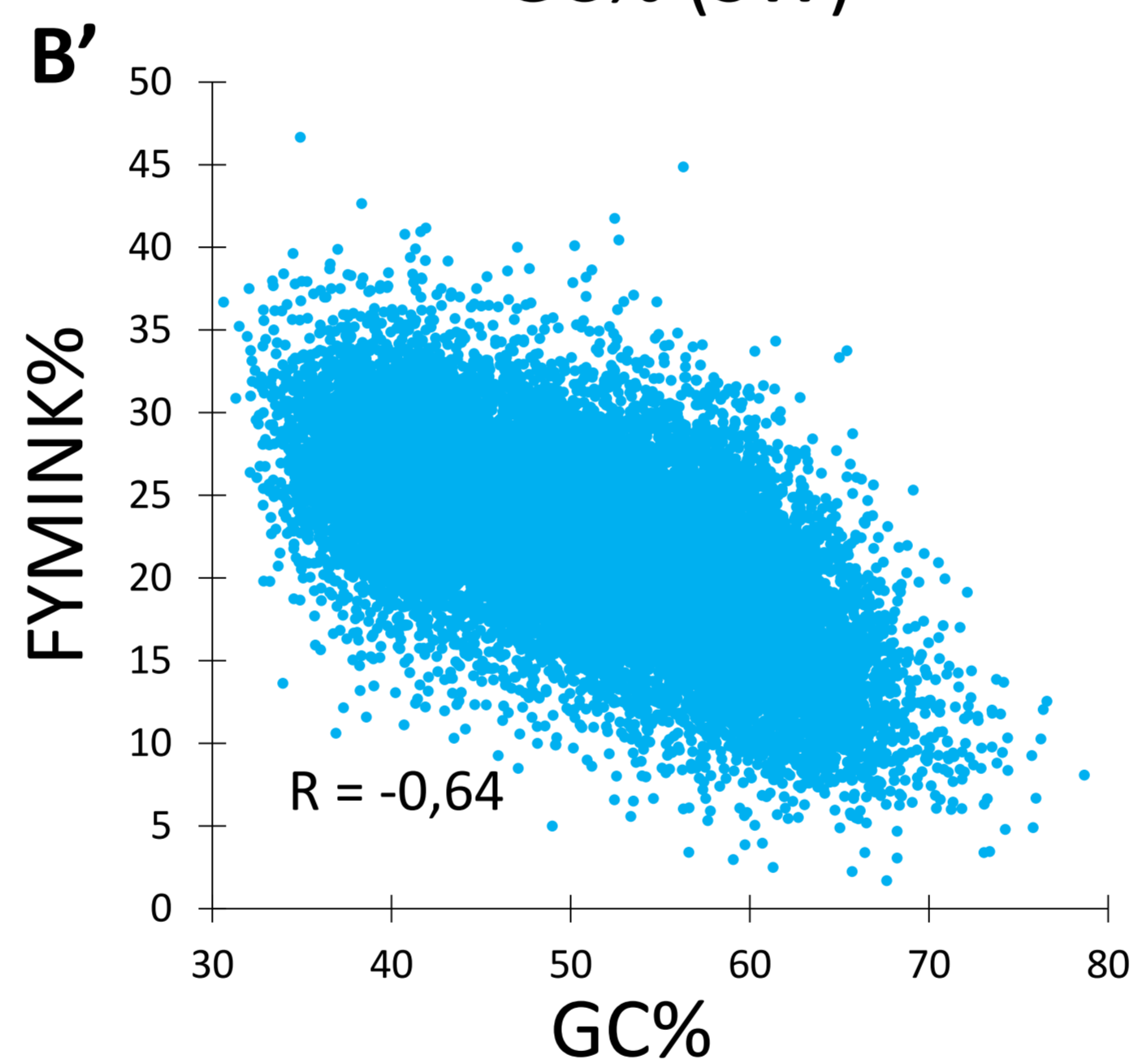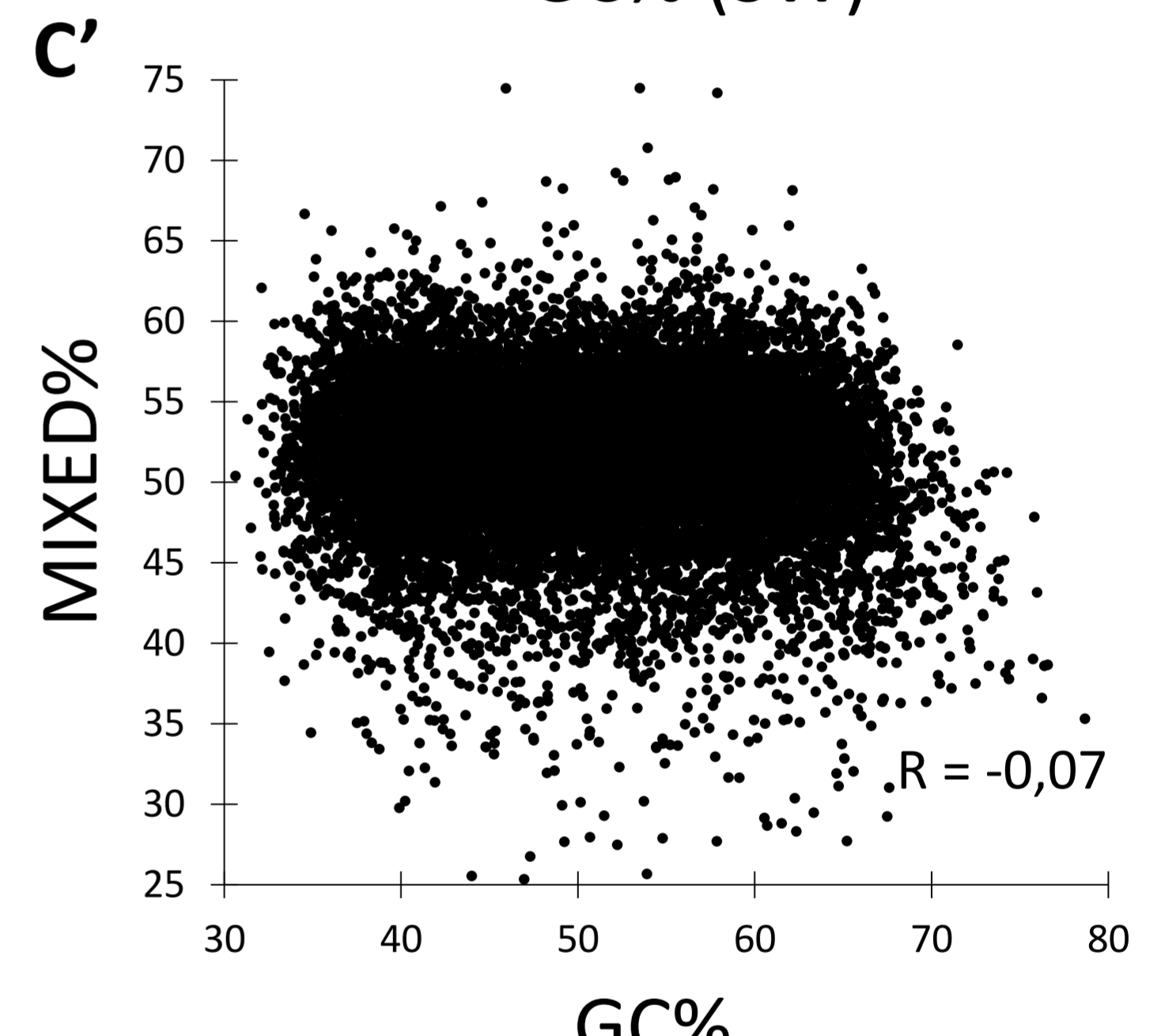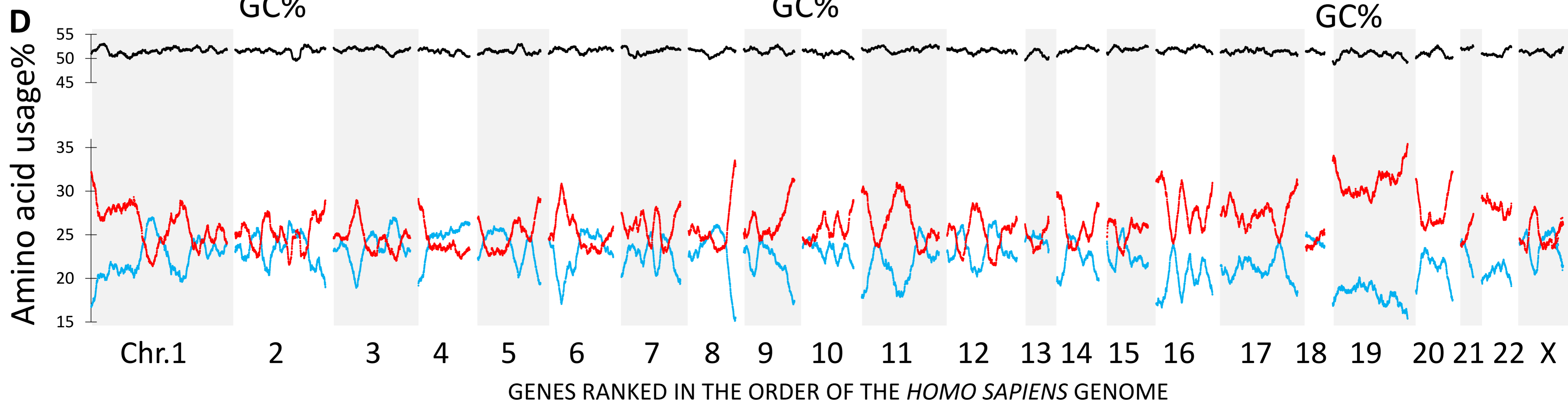

Supplement: Supplementary file 9 — Figure S8. Correlation between GC content of mRNA and GARP% and FYMINK% in encoded proteins. The human data for the 15,824 vertebrate genes were assessed at the level of sliding window 100 means (panels A-C) or values for individual genes of the study (panels A’-C′). In both cases GARP% was positively and FYMINK% negatively correlated to GC content, while the abundance of the other ten amino acids was not influenced by GC content. Panel D shows perfectly mirrored landscapes of GARP% (red; genome-wide average = 26.1%) and FYMINK% (blue; genome-wide average = 22.4%), while the sum of the other ten amino acids (black; genome-wide average = 51.5% of amino acid composition) is not dependent on genomic position. (PDF 7706 kb) [file 12862_2019_1469_MOESM9_ESM.pdf]

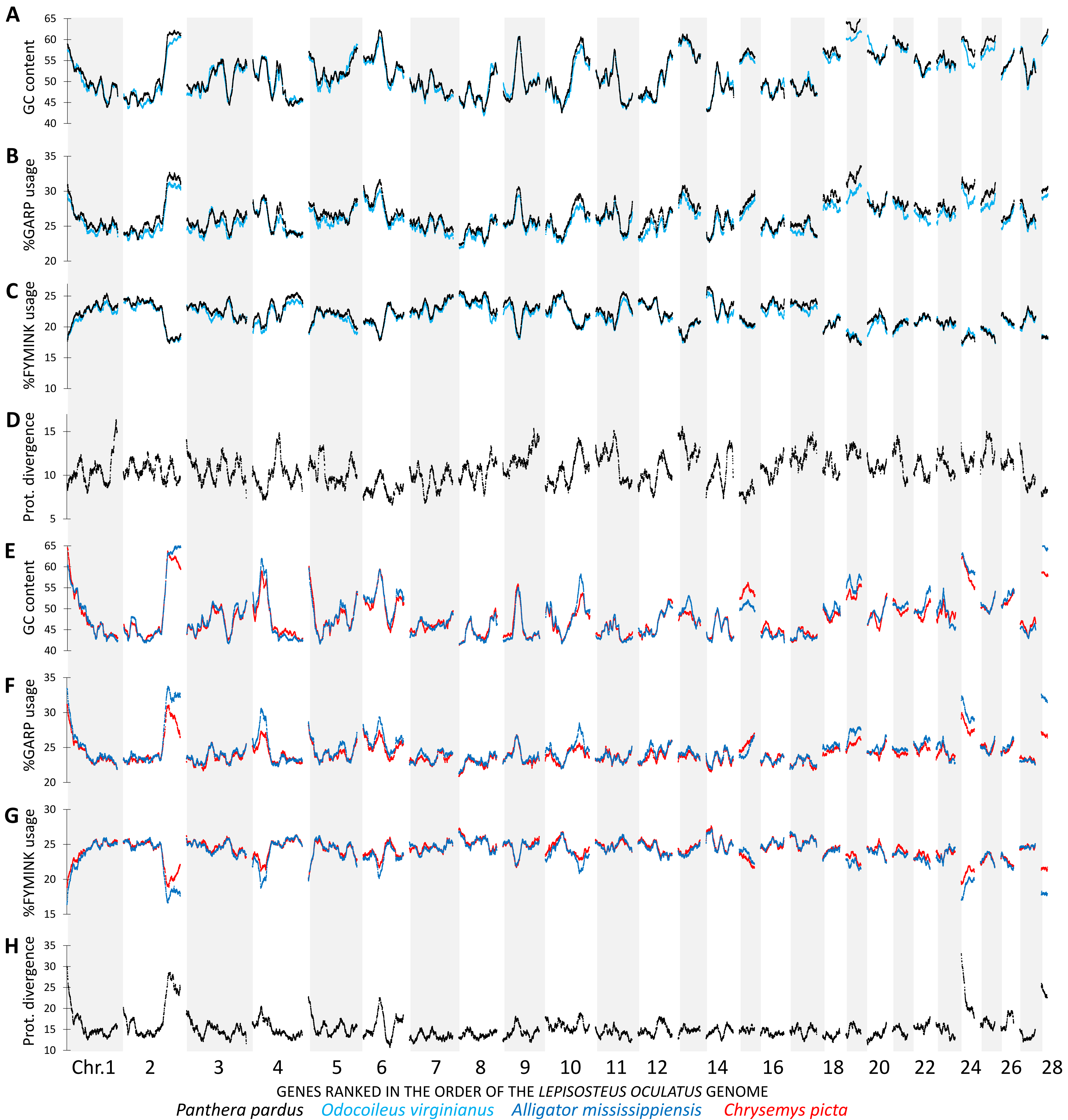

Supplement: Supplementary file 10 — Figure S9. Landscapes of mammals and reptiles, ranked on the spotted gar genome order. Same data as in Fig. 4, but the genes are now ranked on the order of Lepisosteus oculatus (spotted gar) genome. The fluctuations of GC content, amino acid usage and protein divergence correlate as depicted in Fig. 4. R-values for GC content: between Panthera pardus and Odocoileus virginianus texanus (R = 0.97); between Alligator mississippiensis and Chrysemys picta (R = 0.96). (XLSX 14581 kb) (PDF 8947 kb) [file 12862_2019_1469_MOESM10_ESM.pdf]
